# Leveraging Co‐Occurrence to Improve Deep Learning Photo‐Identification in Social Animals

**DOI:** 10.1002/ece3.73552

**Published:** 2026-04-21

**Authors:** Alexander Barnhill, Jared R. Towers, Gary J. Sutton, Tasli J. H. Shaw, Thomas Doniol‐Valcroze, Andreas Maier, Elmar Nöth, Christian Bergler

**Affiliations:** ^1^ Friedrich‐Alexander‐Universität Erlangen‐Nürnberg Pattern Recognition Lab Erlangen Germany; ^2^ Bay Cetology Alert Bay British Columbia Canada; ^3^ Pacific Biological Station, Fisheries and Oceans Canada Nanaimo British Columbia Canada; ^4^ Ocean Wise Vancouver British Columbia Canada; ^5^ Department of Electrical Engineering, Media and Computer Science Ostbayerische Technische Hochschule Amberg‐Weiden Amberg Germany

**Keywords:** co‐occurrence, encounter data, machine learning, photo‐identification, probabilistic fusion, social structure

## Abstract

Photo‐identification underpins individual‐based inference in numerous ecological studies, but scaling it to decades‐long archives remains limited by expert time. Deep learning can accelerate matching, yet most pipelines treat photographs as independent observations and therefore ignore a key aspect of the data collection method: individuals are recorded in structured encounters and often exhibit persistent, non‐random associations. We present a model agnostic, encounter‐level identification procedure that incorporates social context as a deployable probabilistic component. Given per‐image classifier posteriors, we perform log‐linear fusion of three information sources: (i) image‐based probabilities, (ii) global sighting priors (class frequency), and (iii) an encounter‐conditioned context term derived from historical co‐occurrence (log lift). The method operates as lightweight post‐processing and requires no retraining or architectural changes to the image model. Using a longitudinal photo‐identification dataset as a case study (West Coast Transient Bigg's killer whales), we evaluate (a) expert‐assisted settings in which a small number of individuals present in an encounter are known without image‐level labels, and (b) fully automated settings that initialize context from the model's own high‐confidence predictions. On a strict temporal holdout (newest 10%), encounter‐context fusion reduces top‐1 error by ~14%–25% with expert‐assisted seeding; a fully automated variant yields up to ~24% fewer misidentifications once sufficient training history exists, improving Macro‐F1 by +0.088 to +0.104, with minimal computational overhead. Placebo and seed‐corruption controls confirm that gains depend on meaningful co‐occurrence structure and collapse when encounter context is destroyed. By turning encounter structure into a reusable probabilistic component, this work bridges established methods for analyzing animal societies with practical, scalable photo‐identification pipelines. The approach is applicable to any system where individuals are repeatedly observed in groups (e.g., cetaceans, primates, ungulates, camera‐trap bursts) and provides a transparent mechanism to incorporate social context into automated identification.

## Graphical abstract

Photo‐identification of social animals is traditionally manual and time‐intensive, and most deep learning approaches ignore the structured, encounter‐based way in which individuals are observed. We introduce a lightweight, model‐agnostic fusion method that combines image‐level classifier probabilities with global sighting priors and historical co‐occurrence (log‐lift) information at the encounter level, without retraining the underlying model. Using a longitudinal dataset of Bigg's killer whales, we show that incorporating social context reduces top‐1 error by up to ~14%–25% in fully automated settings and substantially more with minimal expert input, with gains disappearing under placebo or corrupted co‐occurrence controls, demonstrating that improvements depend on meaningful social structure.

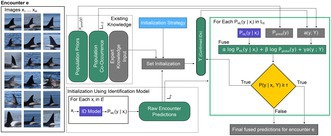

## 1 Introduction

Recognizing and monitoring identifiable individuals plays a key role in ecological research, supporting estimates of survival, reproduction, abundance, movement, and social structure (Alonso et al. 2015; Lebreton et al. 1992). In many species, individuals can be recognized using photographs of natural markings or other persistent phenotypic features, enabling non‐invasive identification (Silvy et al. 2005). Photo‐identification has therefore become a widely adopted tool in ecological monitoring, applied to taxa ranging from amphibians (Gould et al. 2021) and terrestrial mammals (Dixon 2003) to birds (Sherley et al. 2010), sharks (Meekan et al. 2006), and marine mammals (Fujiwara and Caswell 2001).

Photo‐identification has been particularly foundational for cetaceans (Ballance 2018), yielding longitudinal catalogs and datasets of sighting histories spanning multiple decades (e.g., Sears et al. 1990; Towers et al. 2025) and allowing long‐term population research (Hammond et al. 1990; Würsig and Jefferson 1990). Yet the workflow remains heavily dependent on expert judgment (Stevick et al. 2001): field teams must collect, filter, and curate representative images for each individual, and then repeatedly match new photographs to catalogs. As digital photography and storage have improved, many programs have accumulated image archives spanning decades, making curation and matching increasingly rate‐limiting for downstream photo‐identification inference (Swanson et al. 2015).

In parallel, advances in deep learning have reduced human effort in several ecological imaging pipelines, notably in large‐scale camera‐trap workflows where annotation bottlenecks have motivated hybrid human–machine systems (Norouzzadeh et al. 2021; Swanson et al. 2015; Willi et al. 2019). In cetaceans, deep learning efforts have developed to limit the necessity for human involvement by automating key steps such as fin detection, quality filtering, and individual classification (Barnhill et al. 2025; Bergler et al. 2021; Patton et al. 2023). More broadly, multi‐species systems now achieve strong performance across two dozen cetacean species, indicating that deep learning can be robust across heterogeneous catalogs and field conditions (Patton et al. 2023). Despite these advances, the dominant paradigm remains image‐centric: each photograph is treated as an independent observation, and uncertainty due to pose, occlusion, or visual similarity is handled primarily by improving the image model.

This independence assumption is mismatched to how photo‐identification data are collected. Photographs are typically obtained during encounters, defined here as structured field observation events (shared date, location, and photographer) in which one or more individuals are photographed during the same sighting. These encounters constrain the set of plausible identities, and for many social species the probability of co‐occurrence is highly non‐uniform. Many monitoring protocols can generate grouped observations (e.g., encounters, bouts, capture events, camera‐trap bursts, or survey segments), although the prevalence of such structure varies across species and sampling designs.

Encounter structure is particularly informative in species with stable or semi‐stable social associations. In many cetaceans, individuals are repeatedly observed with a limited subset of companions over long time scales, producing non‐random association patterns that underpin much of cetacean social‐network analysis (Whitehead 2008, 2009; Whitehead and Dufault 1999; Weiss et al. 2021). Field researchers also use this context informally during photo‐identification: when imagery is ambiguous, plausible identities are constrained by who else is present and by known patterns of association. Because repeated identifications capture relationships ranging from weak, occasional associations to strong, persistent ones, encounter data provide a natural way to encode ecologically meaningful social structure in identification workflows (Weiss et al. 2019). However, this information is rarely treated as a first‐class, deployable component in modern deep photo‐identification pipelines.

Motivated by this gap, we introduce an encounter‐level context‐assisted identification method that feeds social structure back into recognition without retraining the image model. The fusion framework combines three sources of information available during photo‐identification: (i) image‐based classifier predictions, (ii) global sighting frequencies of individuals, and (iii) encounter‐level co‐occurrence structure derived from historical observations. The goal of the formulation below is to provide an explicit and reproducible description of how these sources of evidence are combined into a single identity score for each image.

This differs from simple encounter‐level aggregation of image predictions (e.g., averaging classifier scores across images in the same encounter). Instead, the proposed method conditions identity scores on the evolving set of identities known or inferred to be present in the encounter and incorporates historical co‐occurrence statistics derived from previous encounters.

In practice, given a per‐image classifier posterior we compute an encounter‐conditioned context prior based on the set of individuals already inferred or hypothesized to be present in the encounter, and fuse image evidence, global sighting priors, and context evidence using a log‐linear model. This yields an interpretable mechanism with explicit weight parameters estimated on validation data that controls how much the final posterior is driven by visual evidence versus ecological context.

Because the method operates purely as post‐processing, it can be attached to existing photo‐identification pipelines and applied retrospectively to archived predictions. Conceptually, the approach mirrors well‐established ideas in classifier fusion and “context as prior,” where multiple weak but complementary evidence sources are combined in a transparent way (Kittler et al. 1998; Rabinovich et al. 2007; Torralba 2003). Our association term is derived from co‐occurrence “lift” (log‐ratio) style scores, which are commonly used to quantify dependence strength, and we clip context evidence to prevent rare co‐occurrence events from dominating predictions (Church and Hanks 1990; Weiss et al. 2019).

Although we demonstrate the method on a long‐term killer whale archive, the procedure is broadly applicable to systems where individuals are repeatedly observed in grouped encounters and per‐image identity probabilities are available. These ingredients arise in many systems with repeated group observations of identifiable individuals (e.g., primates, elephants, social carnivores, colonial seabirds, and camera‐trap bursts). The case study therefore serves to quantify operating regimes and failure modes rather than to define the method's domain.

Several publicly available wildlife re‐identification datasets have recently been developed to support benchmarking of image‐based identification systems (e.g., BelugaID, HappyWhale, HumpbackWhaleID, SeaTurtleID2022). These datasets have been instrumental in advancing computer vision methods for individual recognition. However, the focus of most benchmarks remains the accuracy of image‐level identity predictions, while encounter‐level structure, such as information about which individuals were observed during the same field sighting, is often incomplete, inconsistently defined, or unavailable. In addition, the proposed method exploits persistent, non‐random association structure among individuals, meaning that meaningful evaluation requires datasets in which encounter groupings reflect genuine social relationships rather than incidental spatial or temporal overlap. Because the method relies on encounter‐level grouping to estimate historical co‐occurrence structure without temporal information leakage, we demonstrate the approach using a long‐term killer whale photo‐identification archive in which encounters are explicitly recorded. The aim of this study is therefore not to propose a new image‐recognition architecture or benchmark dataset, but to evaluate whether encounter‐conditioned contextual information can improve identification performance for an existing classifier.

Using a longitudinal dataset of the West Coast Transient population of Bigg's killer whales as a case study, we quantify when and how encounter context helps identification under two practical deployment modes: (i) expert‐assisted inference, where a small number of individuals are known to be present in an encounter without image‐level annotation; and (ii) fully automated inference, where context is initialized using the model's own high‐confidence predictions. To reflect realistic “future deployment” and to prevent leakage, we evaluate on a strict temporal holdout and construct priors and co‐occurrence artifacts from training encounters only, consistent with recommendations for structured (temporally dependent) ecological validation (Roberts et al. 2017). Overall, we show that encounter context provides a deployable mechanism to re‐rank visually plausible candidates, bridging established methods for analyzing animal societies with scalable, modern photo‐identification workflows.

## 2 Methods

### 2.1 Study System and Dataset

#### 2.1.1 Study Population and Image Archive

The West Coast Transient population of Bigg's killer whales (*Orcinus orca rectipinnus*), found in the northeastern Pacific Ocean, are used as a case study here due to their longevity, social nature, and long‐running focus as a threatened population (Baird 2000; Bigg 1982; Nielsen et al. 2023; Towers et al. 2025). The photos of this population used for this study were collected from 2011 to 2021 and include a total of 405 individuals present in 3818 encounters, with an average number of individuals per encounter of 4.5 (min: 1, max: 30, std: 3.5). The photos are organized by encounter, which includes a distinct location name, date, and photographer.

#### 2.1.2 Temporal Splitting and Leakage Control

To ensure a realistic evaluation of historical data, encounters are ordered chronologically so that all validation and test encounters occur strictly after the training encounters. As a result, both the prior sighting probabilities and the co‐occurrence term are computed only from the training split, preventing information from future encounters from leaking into training or evaluation and biasing the results.

#### 2.1.3 Closed‐Set Core

From the full archive (405 individuals, 3818 encounters), we restrict training to single‐fin, singly‐labeled crops and then to a closed‐set core of 104 consistently observed individuals, yielding 53,724 images across 3175 encounters. Splits are constructed so that these same 104 classes appear in every train/validation/test partition in all training fractions. This design enforces a closed‐set identification setting and avoids confounding effects where some training fractions would otherwise be evaluated on identities that never occur in their training history. As a result, any performance differences can be attributed to the amount of historical data and the initialization strategy, rather than to changes in the underlying label set. We note that this focuses the analysis on a well‐sampled subset of the population; extending the approach to rarer or newly‐appearing individuals is an important direction for future work.

### 2.2 Baseline Identification Model

#### 2.2.1 Architecture and Training Procedure

The baseline for the application of historical data relies first on the underlying deep learning model. In this case, the baseline model architecture is always the same, namely an EfficientNet B0 (Tan and Le 2019) model with a classification head with an output size corresponding to the number of individuals in the training/evaluation data. The baseline architecture is intentionally kept simple, as the goal of this study is to evaluate the effect of contextual fusion rather than to optimize image‐recognition performance. Importantly, the proposed fusion framework is model‐agnostic and operates only on the probability distributions produced by the classifier.

#### 2.2.2 Per‐Image Posterior Definition

**FIGURE 1:**
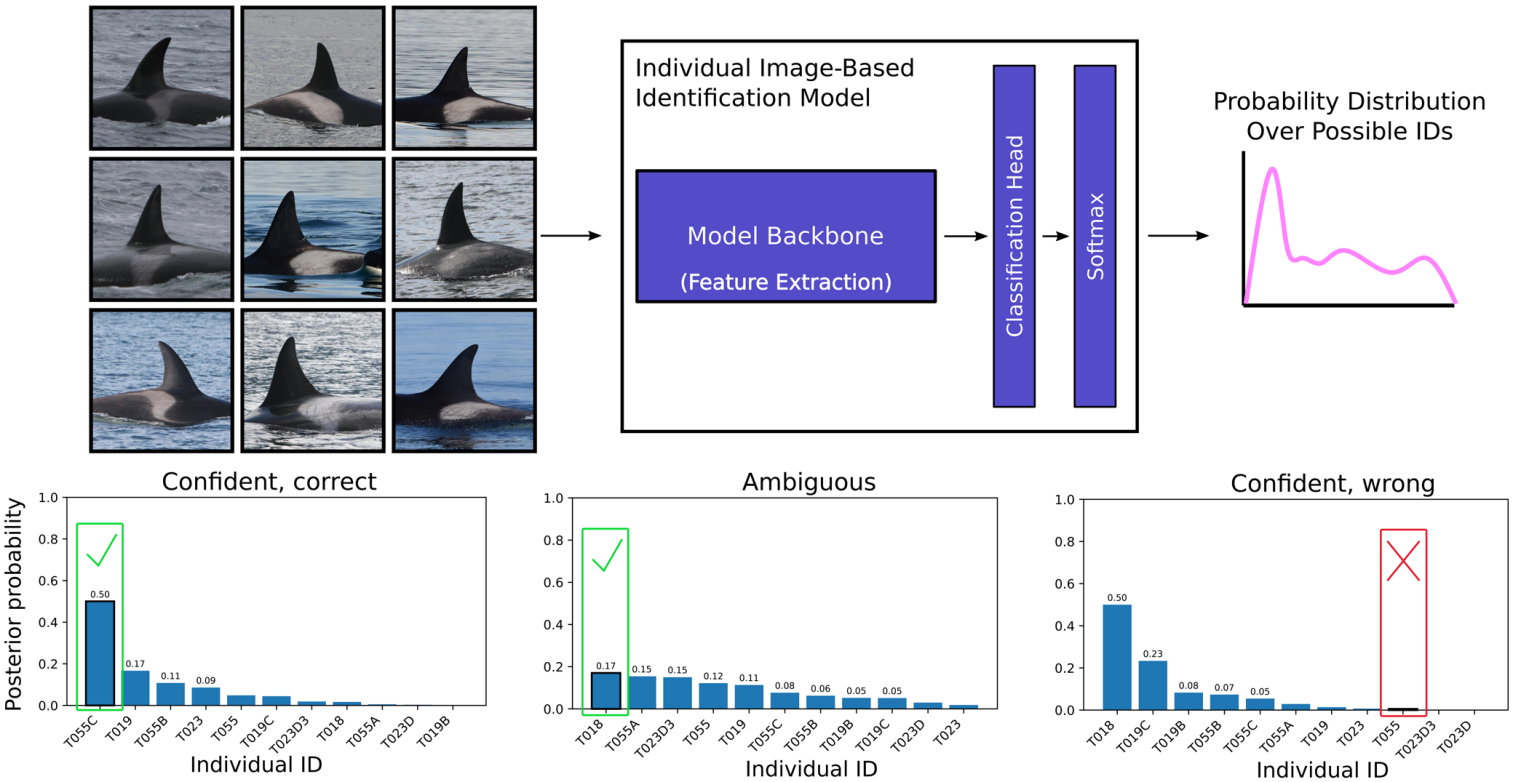
Demonstration of the baseline classifier and generation of per‐image softmax posteriors. Examples illustrate confident, ambiguous, and confidently incorrect outputs. Images reproduced with permission from data contributors.

The output of our identification model z for image $x_{i}$ is a vector containing the logit distribution over all possible N individuals. These logits are converted to probability space by applying the softmax function. This yields a definition for our model posterior as $P_{\mathrm{ML}}\left(y|x\right)=\text{Softmax}\left(z\left(x\right)\right)$. This process is visualized conceptually in Figure 1, where it is also shown how probability distributions can shift based on how “confident” the model is in its prediction.

### 2.3 Social Context Statistics From Training History

#### 2.3.1 Global Sighting Priors

Using training encounters only, we compute a global sighting prior for each individual. Let E denote the set of training encounters. For an encounter e∈E, a confirmed sighting of individual i contributes weight 1, and an uncertain sighting (marked “?” by experts) contributes a weight of 0.5. Let $n\left(i\right)$ be the resulting weighted count of encounters in which i appears. We define the global prior probability of observing i in a randomly selected training encounter as $P_{\text{global}}\left(i\right)=\frac{n\left(i\right)}{\left|E\right|}$.

These priors capture heterogeneous sighting frequencies across individuals and are used as a deployable prior term during encounter‐level inference.

#### 2.3.2 Co‐Occurrence Log Lift Matrix

**FIGURE 2:**
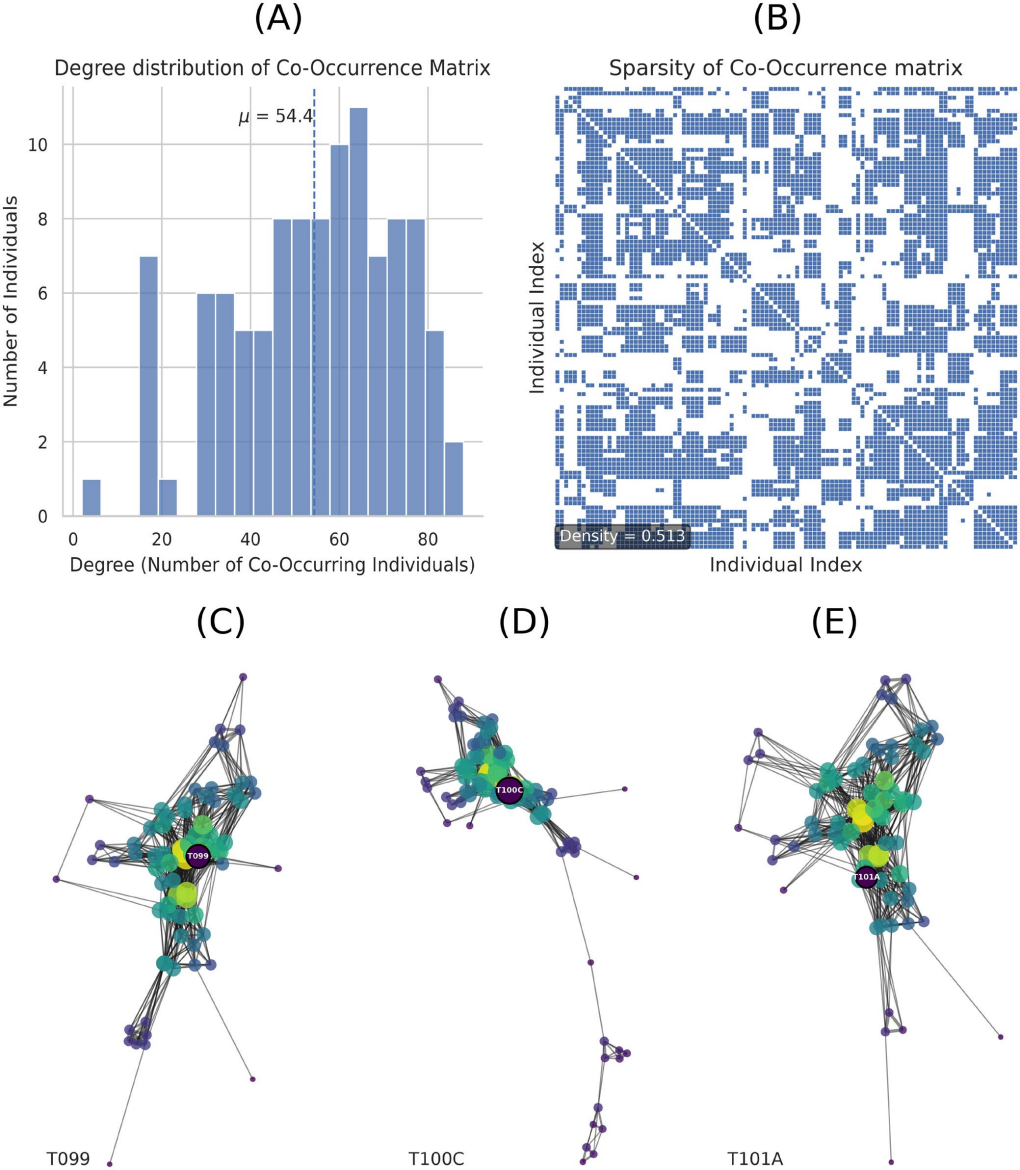
Bigg's killer whale co‐occurrence structure. (A) Degree distribution of the co‐occurrence graph (number of distinct companions per individual). (B) Sparsity of the 405×405 co‐occurrence matrix; non‐zero entries indicate at least one historical co‐occurrence. (C–E) Ego‐networks for three high‐degree individuals; node size reflects co‐occurrence frequency with the focal individual and edge width reflects pairwise co‐occurrence weight.

We next construct an N×N association matrix that quantifies how strongly pairs of individuals co‐occur in training encounters. For each encounter e∈E, we add a weighted contribution to each pair $\left(i,j\right)$ present in that encounter, using the same confirmed/uncertain weights as above. When both identities are uncertain the pair receives weight 0.25, reflecting the product of the individual encounter weights. Let $n\left(i,j\right)$ denote the resulting weighted count of encounters in which i and j co‐occur. We define $P\left(i,j\right)=\frac{n\left(i,j\right)}{\left|E\right|}$, and compute the log lift association score $L_{i,j}=\log\frac{P\left(i,j\right)+\epsilon }{\left(P\left(i\right)+\epsilon \right)\left(P\left(j\right)+\epsilon \right)}$, where ϵ>0 ensures numerical stability (here $\epsilon =1^{-6}$). We clip log lift values to $\left[-6,6\right]$ to prevent extremely rare co‐occurrence events from producing disproportionately large context scores. Since the context score is an average over encounter members and γ is selected on validation data, the method cannot rely on unbounded social priors to override visual evidence. The connectivity as well as the sparsity of the co‐occurrence structure for the study population is summarized in Figure 2, as well as three examples displaying the connectivity of certain individuals.

#### 2.3.3 Encounter‐Conditioned Context Prior

Usage of the co‐occurrence information in any fusion process requires some degree of knowledge about who is present. This list of individuals confirmed or otherwise suspected to be present Y in encounter e therefore directly affects the contextual prior. This associative term for candidate y is defined as

(1) $$a\left(y;Y\right)=\frac{1}{\left|Y\right|}\sum_{i\in Y}L_{i,y}\;,\;\text{if}\;\left|Y\right|>0,\;\text{else}\;0$$

It should be noted that if Y=ϕ, no contextual information is available and the association term contributes 0, so fusion reduces to combining the image posterior with the global prior only.

**FIGURE 3:**
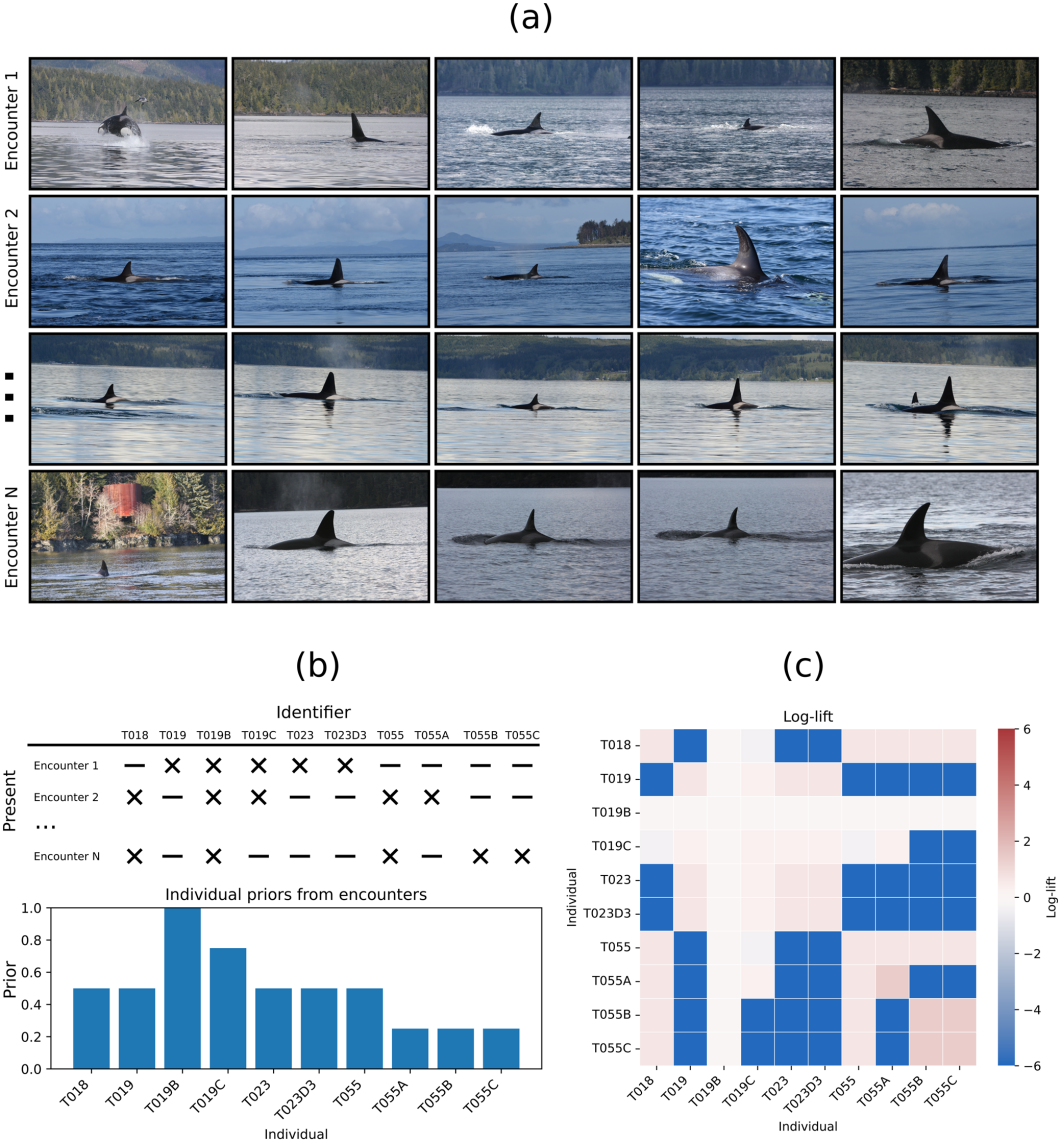
Example encounter structure and derived context artifacts. (A) Four hand‐selected encounters (rows), each shown with five cropped dorsal‐fin images from photographs taken during the same encounter. (B) Global sighting priors (fraction of encounters in which each individual appears). (C) Co‐occurrence log lift matrix (prevalence‐corrected), computed as the log ratio between observed pairwise co‐occurrence and the expectation under independent marginal frequencies. Positive values indicate above‐chance co‐occurrence; negative values indicate avoidance. In this subset, log lift highlights stable associations such as T023–T023D3 and the T055 matriline (notably T055B–T055C). Images reproduced with permission from data contributors.

This associative term forms the basis of all fusion experiments to follow. An example of the creation of these contextual priors from a series of encounters is shown in Figure 3.

#### 2.3.4 Initialization Strategies

Encounter context requires an initial hypothesized membership set Y. We evaluate four representative strategies. Null Set uses Y=ϕ. Random‐n Known seeds Y with *n* ground‐truth encounter members (identity‐only, no image‐level labels), representing expert‐assisted confirmation. Top‐*n* Unknown seeds Y with the n unique identities with highest baseline confidence within the encounter (self‐seeding). Random‐*n* Unknown samples *n* unique identities from the set of baseline argmax predictions appearing in the encounter, representing noisy/self‐seeding. Additional variants are described in Appendixes S1–S3. Unless otherwise stated, we use $n\in \left\{1,2,3\right\}$ unique identities for strategies requiring n identities and ignore duplicates. Full definitions of all initialization strategies, including additional exploratory variants, are provided in Appendix S1.

#### 2.3.5 Fusion Rule

With the raw model predictions, and the list of confirmed individuals, the fusion process can be represented as follows:

(2) $$\begin{array}{cc} \log P_{\text{fuse}}\left(y|x,Y\right)= & \alpha \text{log}P_{\mathrm{ML}}\left(y|x\right)+\beta \text{log}P_{\text{global}}\left(y\right)\\ & +\gamma a\left(y\;;\;Y\right)-\text{logZ}\left(x,Y\right), \end{array}$$

where α,β,γ∈ℝ≥0 control the influence of the image model, global sighting frequency, and co‐occurrence structure, respectively, and $\text{logZ}\left(x,Y\right)$ is the log normalizing constant ensuring $\sum_{y}P_{\text{fuse}}\left(y|x,Y\right)=1$.

#### 2.3.6 Encounter‐Level Inference Using Social Context

Let encounter e contain images $X_{e}=\left\{x_{1},\ldots x_{M}\right\}$. For each image x the baseline classifier provides a posterior $P_{\mathrm{ML}}\left(y|x\right)$ over the N candidate identities $y\in \left\{1,\cdots ,N\right\}$, which are all stored for downstream use. Let $P_{\text{global}}\left(y\right)$ denote global sighting priors computed from training encounters only, and let a be the encounter‐conditioned association score from Equation (1), where Y is the current set of confirmed (or hypothesized) identities present in encounter e.

To mimic typical “easy‐first” workflows and to reduce error propagation, we process images in descending order of baseline confidence $\max_{y}P_{\mathrm{ML}}\left(y|x\right)$ when evaluating strategies which rely on posterior model probability. For randomized strategy variants, we instead shuffle the image order.

For each image x and current confirmed set Y, we compute the fused posterior via Equation (2). The fused prediction is therefore defined as $\hat{y}\left(x\right)=\arg\max_{y}P_{\text{fuse}}\left(y|x,Y\right)$.

**FIGURE 4:**
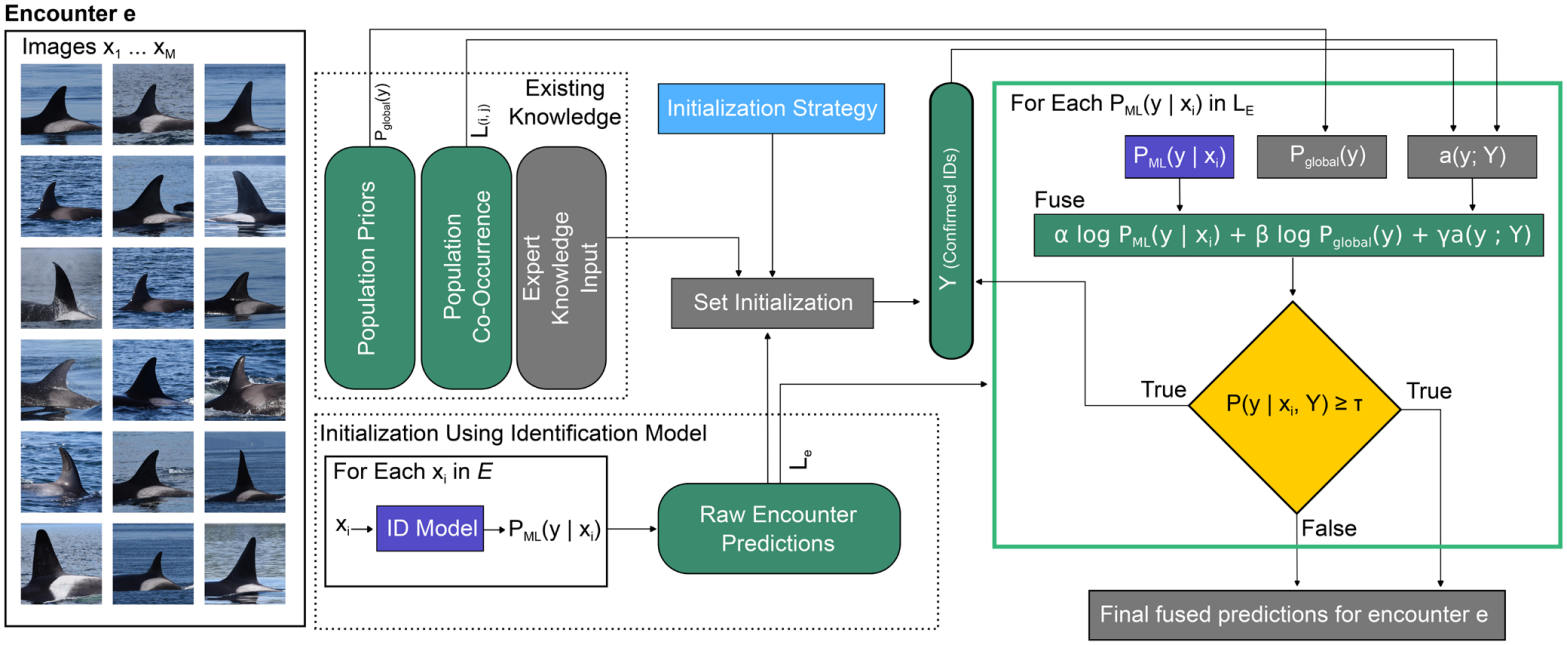
Encounter‐level co‐occurrence fusion pipeline. Per‐image classifier posteriors are updated using priors and co‐occurrence conditioned on an evolving encounter membership set. Images reproduced with permission from data contributors.

We perform a single pass through the encounter. After computing $P_{\text{fuse}}\left(\cdot |x,Y\right)$, we update the confirmed set if the fused confidence exceeds a threshold τ, which here is set to 0.95 for all experiments to favor high‐precision additions to Y. Duplicate identities are ignored, so Y contains unique individuals only. When Y=Φ, Equation (1) yields $a\left(y;Y\right)=0$ for all y, and Equation (2) reduces to combining the image posterior with the global prior. We use a single‐pass update (no repeated re‐processing of earlier images) to limit reinforcement effects. An example of this procedure is illustrated in Figure 4.

### 2.4 Optimization of Fusion Weights

The process for the selection of the fusion weights $\left\{\alpha ,\beta ,\gamma \right\}$ as seen in Equation (2) is described below. For the sake of comparison, a discrete grid‐search was also performed which yields similar optima (Table S1). Both procedures use the same encounter‐level inference pipeline and differ only in how weights are chosen on the validation split. Complete weight search procedures, including the grid‐search protocol, are detailed in Appendix S3. Additional figures and full results tables are collected in Appendixes S4 and S5, respectively.

#### 2.4.1 Model‐Selection Criterion (Lexicographic)

**FIGURE 5:**
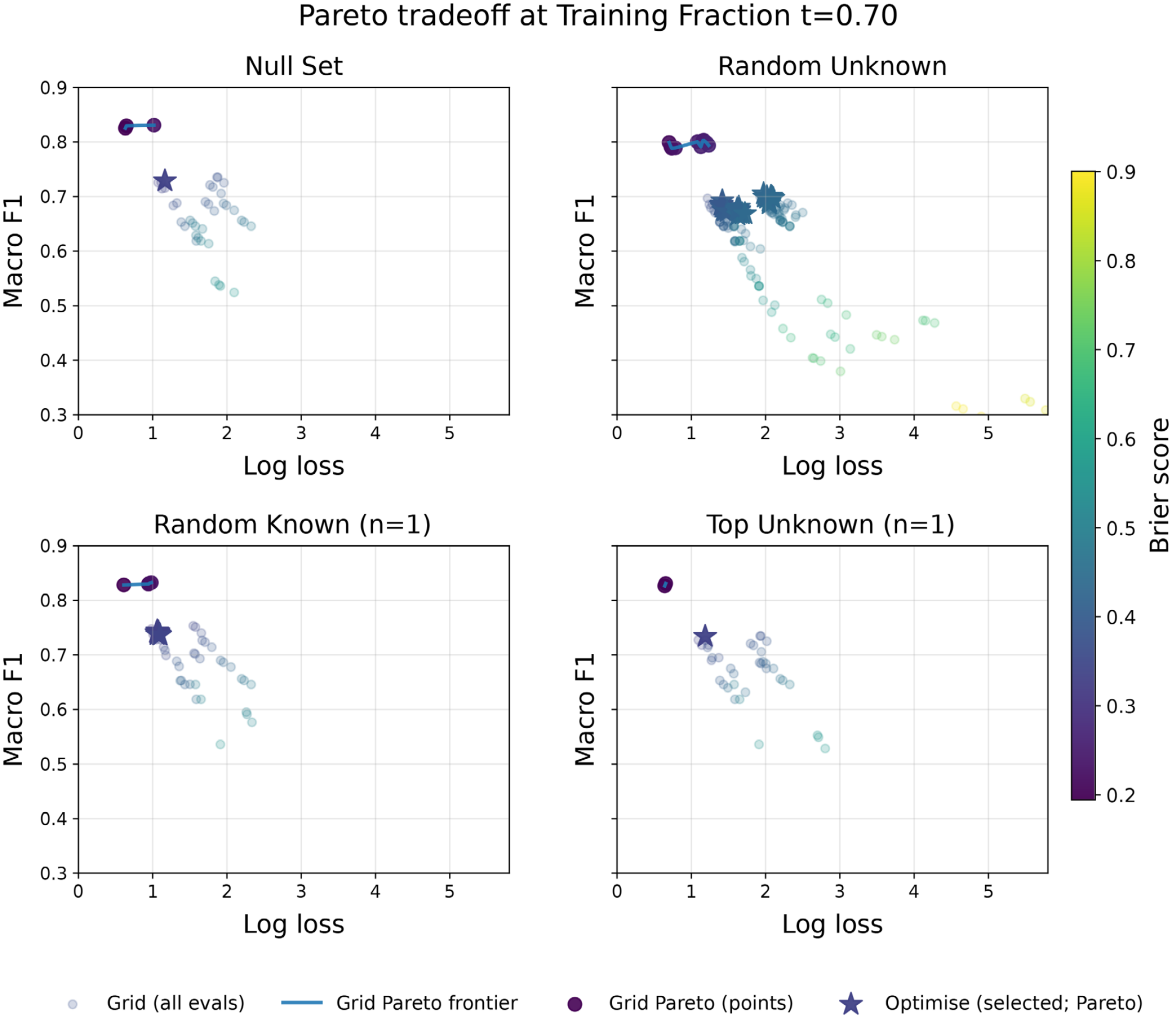
Pareto trade‐offs for fusion‐weight selection at training fraction t=0.7. Each panel shows one initialization strategy (Null Set, Random Unknown, Random Known (*n*=1), Top Unknown (*n*=1)). Semi‐transparent points show all evaluated weight configurations; the connected curve indicates the corresponding Pareto frontier (higher macro‐F1, lower log loss and Brier score). Stars mark the weights selected by the staged optimization procedure using the lexicographic criterion (maximize macro‐F1; break ties by log loss, then Brier); outlines indicate cases where the selected point is not Pareto‐optimal under the evaluated set. Point color encodes Brier score (shared color scale across panels), illustrating the calibration–discrimination trade‐off. Axes are shared across panels to enable direct comparison.

Weights were selected on the validation split by maximizing mean macro‐F1; ties were broken by lower log loss, then lower Brier score. In addition, we retain all evaluated configurations and visualize the calibration‐discrimination trade‐off using Pareto frontiers over these metrics (Figure 5; Figure S3).

#### 2.4.2 Staged Optimization of Fusion Weights

Our procedure uses a two‐stage search on the validation split.

Stage A—Optimize $\left\{\alpha ,\beta \right\}$, with γ=0.

We first select $\left\{\alpha ,\beta \right\}$ while disabling the co‐occurrence term (γ=0), so that fusion combines only image posterior and global priors. Depending on the configured mode, $\left\{\alpha ,\beta \right\}$ are chosen either by grid search or by a lightweight coordinate‐wise hill‐climb around the best coarse candidate.

Stage B—Optimize γ, with $\left\{\alpha ,\beta \right\}$ fixed.

With $\left\{\alpha ,\beta \right\}$ fixed to the Stage A optimum, we sweep $\gamma \in \left\{0.0,0.5,1.0,2.0\right\}$ and select the best value under the same lexicographic criterion.

#### 2.4.3 Data Leakage Control During Weight Selection

Throughout, co‐occurrence statistics and sighting priors are computed from training encounters only (and remain fixed), and baseline classifier outputs are held fixed; thus optimization affects only how these existing probability sources are combined.

For stochastic initialization strategies (e.g., Random*), we run 10 random seeds and report mean ± standard deviation (STD); deterministic strategies are run once.

#### 2.4.4 Ablations: Separating Image, Priors, and Co‐Occurrence Contributions

To disentangle the contributions of (i) the image‐based classifier posterior, (ii) global sighting priors, and (iii) co‐occurrence log lift, we run a targeted ablation suite in which we hold the inference update rule and threshold fixed; ordering/initialization are varied only to include or exclude ML guidance. Full ablation definitions are provided in Supporting Information.

For ablations involving co‐occurrence, we sweep $\gamma \in \left\{0.0,0.5,1.0,2.0\right\}$ while holding $\left\{\alpha ,\beta \right\}$ fixed per ablation definition. Formal definitions of each ablation configuration are given in Appendix S2.

#### 2.4.5 Sensitivity Analysis: Robustness to Noisy Encounter Seeding

Because the fusion update is explicitly conditioned on the set of identities assumed to be present in an encounter, its gains should depend on the *quality* of that initial membership information. To quantify this dependence, we ran a targeted sensitivity analysis in which we intentionally corrupt the encounter seed set while holding the inference update rule, fusion weights, and threshold fixed. Concretely, we focus on the four headline initialization strategies and evaluate two representative training regimes (t=0.3, t=0.7) on the fixed future test split. For each encounter, we first generate an initial set $Y_{0}$ using the chosen strategy, then apply controlled corruption to produce a perturbed seed set ${\tilde{Y}}_{0}$, which is used for the subsequent single‐pass fusion update. Corruption is applied probabilistically but is seeded deterministically (by global seed and encounter identifier) to ensure reproducibility; thus, variability (±SD) reflects stochastic initialisation (Random*) rather than repeated corruption draws. Deterministic strategies (Null Set, Top Unknown) are therefore evaluated once under this fixed corruption realization.

We hold the fusion update rule, weights, and decision threshold fixed, and vary only the initialization strategy and the amount/type of seed corruption. For each encounter, corruption is applied stochastically with probability p (reported as $p\in \left\{0,0.1,0.2,0.4,0.6\right\}$). We consider three noise modes: drop, where each seed label is independently removed with probability p (simulating missing or withheld encounter membership); swap, where each seed label is independently marked for replacement with probability p and replaced by a randomly drawn identity from the label universe, rejecting draws already present in ${\tilde{Y}}_{0}$ (simulating incorrect labels supplied as encounter members); and add, where a number of “decoy” labels from the label universe are sampled as $k∼\text{Binomial}\left(n_{\text{init}},p\right)$ and added to the seed set (simulating extra spurious members being included). We report performance as mean ± SD across random seeds for stochastic initializers (single seed otherwise). This sensitivity suite directly tests whether the gains from co‐occurrence fusion persist when expert‐provided encounter membership is incomplete, noisy, or contaminated by plausible labeling errors. For this evaluation we fix $n_{\text{init}}=1$ to reduce complexity, thus, p is the probability that the single seed is missing (drop)/incorrect (swap)/contaminated (add). With $n_{\text{init}}=1$, the add mode introduces at most one decoy identity per encounter (since $k∼\text{Binomial}\left(1,p\right)$).

#### 2.4.6 Placebo Co‐Occurrence and Weight‐Selection Controls

Because gains from encounter‐conditioned fusion could arise either from meaningful co‐occurrence structure or from the fusion rule exploiting any structured matrix (including artifacts of label frequency), we ran two final control experiments that isolate the role of the co‐occurrence signal and the role of weight tuning.

##### 2.4.6.1 Placebo Co‐Occurrence Experiment (Fixed Weights)

We constructed a placebo co‐occurrence prior by breaking the association structure between identities while preserving the overall marginal statistics of the training data. Concretely, for each training fraction t, we first computed the global sighting priors and the encounter‐level co‐occurrence counts from the training encounters only, using the same uncertain‐ID weighting scheme (confirmed IDs weight 1.0, uncertain IDs weight 0.5). We then generated a placebo encounter table by shuffling identity membership across encounters while preserving the encounter‐size distribution (i.e., the number of identities per encounter) and the per‐identity marginal prevalence induced by the training encounters. From this placebo encounter table we recomputed the co‐occurrence matrix and derived the corresponding log lift matrix using the same smoothing and normalization as in the real condition. This produces a matrix with comparable scale and density to the true log lift but without biologically meaningful association structure.

We then evaluated the full inference pipeline under fixed fusion weights $\left(\alpha ,\beta ,\gamma \right)=\left(1.0,0.5,1.0\right)$, holding the update rule, decision threshold, and baseline classifier outputs fixed. We compare performance under (i) the true training‐derived log lift and (ii) the placebo log lift, using the same four headline initialization strategies: Top Unknown (*n* = 1), Random Unknown (*n* = 1), Random Known (*n* = 1, 2, 3), and the Null Set. Stochastic strategies are run for 10 random seeds; deterministic strategies are run once. This experiment tests whether the improvements attributed to co‐occurrence persist when the co‐occurrence term is replaced by a structure‐preserving but association‐destroying control.

##### 2.4.6.2 Weight‐Selection Control Under Placebo and True Co‐Occurrence (Optimization Runs)

To test whether the weight‐selection procedure can recover the appropriate reliance on the co‐occurrence term (i.e., down‐weighting it when it is uninformative or harmful), we repeated fusion weight optimization under both the true and placebo co‐occurrence conditions. Weight selection is carried out on the validation split using the staged procedure described above. Throughout this optimization, priors and co‐occurrence statistics are computed from training encounters only and remain fixed; only the combination weights vary. Together with the fixed‐weight placebo experiment, this control disentangles (i) whether co‐occurrence gains depend on genuine encounter association structure, and (ii) whether the validation‐driven optimization procedure suppresses reliance on the co‐occurrence term when that structure is removed.

### 2.5 Evaluation Protocol and Metrics

In order to explore the effect of changing amounts of data on the fusion process, we vary the size of the training set, and therefore the amount of data which is used in the model training and calculation of the priors. The training fraction is defined as $t\in \left\{0.10,0.30,0.50,0.70\right\}$, and the test set is fixed as the last 10% of the data. In this regime, the test set always corresponds to the same “future” portion of the time series, while the amount of historical data available to build the classifier, priors, and co‐occurrence matrix changes. This design allows us to directly assess how additional historical data affect the usefulness of co‐occurrence information in a realistic deployment scenario.

For each training fraction t, we partition the full ordered sequence of encounters into three contiguous blocks: (1) training: the earliest t fraction of encounters, (2) validation: the next $\left(1-t\right)/2$ fraction of encounters, (3) holdout: the remaining $\left(1-t\right)/2$ fraction of encounters, which represent the most recent portion of the series for that t.

The fixed future test set is identical across all values of t and is the only split used for reported evaluation. Because the holdout block always extends to the end of the series of encounters, it can overlap the fixed future test set. This overlap does not affect reported results: holdout is only used during deep model training as an internal monitoring split and is never used for model selection, weight selection, or reporting. Model selection (for both the baseline classifier and the fusion weight selection) is performed exclusively on the validation block.

For each split configuration, we evaluate the test data in two ways: (1) directly using the baseline deep learning model, and (2) using the same model but with its predictions refined by the priors and co‐occurrence matrix.

We report top‐1 accuracy, macro‐F1, macro‐precision, and macro‐recall for identification performance; to preserve readability, precision and recall for the headline strategies are provided in Table S6. In addition, we report relative accuracy error reduction (rAER), defined as

$$\text{rAER}=\frac{\left(1-\text{Accurac}y_{\text{baseline}}\right)-\left(1-\text{Accurac}y_{\text{method}}\right)}{\left(1-\text{Accurac}y_{\text{baselline}}\right)}=\frac{A_{\text{method}}-A_{\text{baseline}}}{1-A_{\text{baseline}}}$$

In order to contextualize accuracy changes with respect to the baseline (ML‐only) approach:

For probabilistic calibration we report log loss and Brier score. Unless otherwise stated, results are reported on the fixed future test set and summarized as mean ± SD across random seeds for stochastic strategies.

## 3 Results

### 3.1 Weight Selection by Optimization

Table 1 reports headline strategies at the largest training fraction (t=0.7). Under staged optimization, Random Known (N=1) achieved a macro‐F1 of 0.739, while the Null Set reached 0.729. Fully automated strategies were competitive but more sensitive to seed quality (e.g., Random Unknown: 0.697; Top Unknown: 0.734). Fusion weights were stable across regimes and similar to the discrete grid‐search optima (Table S1). Gains were largest when baseline predictions were uncertain and degraded most under incorrect (swap) initialization, while remaining robust to missing seeds (drop) and spurious identities (add), consistent with context amplifying errors when encounter membership is mis‐specified. Macro‐precision and macro‐recall showed the same qualitative pattern as macro‐F1 across the headline strategies (Table S6), indicating that improvements were not driven solely by either reduced omission or reduced commission errors.

**TABLE 1:** Performance of key initialization strategies under the staged optimization weight‐selection procedure at training fraction t=0.7.

Strategy	*n*	α	β	γ	Macro F1	Accuracy	Log Loss	Brier	rAER	Coverage
Baseline	—	—	—	—	0.651	0.682	—	—	—	—
Random Known	3	2	2	2	0.726 (0.001)	0.747 (0.001)	1.870 (0.008)	0.414 (0.001)	0.203 (0.002)	0.982
Random Known	2	2	2	2	0.727 (0.001)	0.747 (0.001)	1.874 (0.010)	0.412 (0.002)	0.205 (0.003)	0.968
Random Known	1	1	0.5	1	0.739 (0.002)	0.761 (0.002)	1.075 (0.016)	0.335 (0.004)	0.247 (0.008)	0.88
Top Unknown	1	1	0	1	0.734	0.760	1.187	0.335	0.244	0.831
Null Set	—	1	0	1	0.729	0.760	1.165	0.334	0.243	0.815
Random Unknown	—	1.75	0	1.5	0.697 (0.005)	0.725 (0.003)	2.040 (0.043)	0.435 (0.004)	0.135 (0.010)	0.855

*Note:* For each strategy we report the initial set size n and the learned fusion weights $\left(\alpha ,\beta ,\gamma \right)$. Metrics are shown as mean (±SD) across random seeds for stochastic strategies; deterministic strategies evaluated once are reported as a single value. rAER denotes relative accuracy error reduction with respect to the ML‐only baseline. Coverage is the fraction of ground‐truth encounter members included in the final inferred identity set Y. Weights are selected on the validation split using the lexicographic criterion described in Section 2.

**FIGURE 6:**
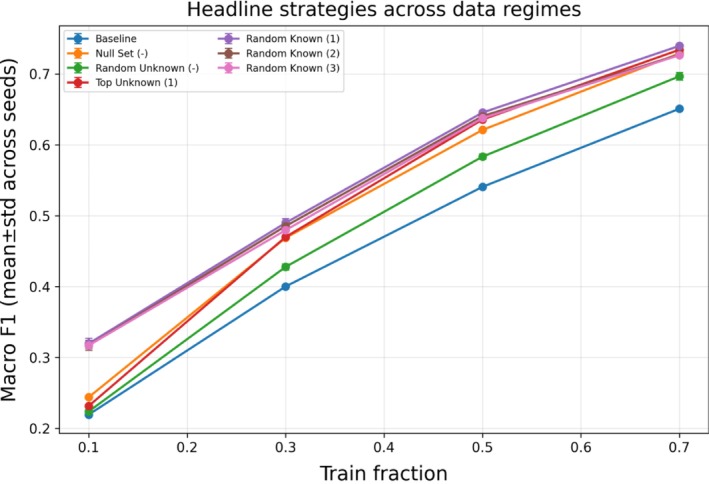
Performance of several headline strategies across all training fractions on the fixed future test set (newest 10% of encounters). Results are shown relative to the ML‐only baseline. Macro‐F1 is reported as mean (±SD) across 10 random seeds for stochastic initialization strategies; deterministic strategies are run once.

Figure 5 shows the Pareto tradeoff for this training fraction for these four strategies. Furthermore, the tendency of the headline strategies' performance across all training fractions, seen in Figure 6, demonstrates that these strategies consistently outperform the baseline (ML‐only) classifier, with respect to macro‐F1 score. The optimizer‐selected weights were similarly consistent with the grid‐search maximizers and often lay on or near the Pareto frontier (Figure 5; Tables S2 and S3). Full results showing macro‐F1 across both the grid search and stated optimization approaches for all initialization strategies and training fractions are shown in Figure S1 (grid search) and Figure S2 (staged optimization).

### 3.2 Ablation: Co‐Occurrence and Priors in Isolation

We evaluated ablations in which the fused score excluded the image‐model term (α=0), leaving only priors (β) and co‐occurrence/loglift (γ) components (Table S4). Under ORACLE initialization (all ground‐truth identities provided at encounter initialization), accuracy ranged from 0.047 at *t* = 0.10 to 0.092 at *t* = 0.70, and macro‐F1 ranged from 0.036 to 0.059. Across training fractions and reported strategies, ΔMacro‐F1 and ΔAccuracy relative to the ML‐only baseline were negative.

In the ML‐only ablation (α = 1, β = 0, γ = 0), ΔMacro‐F1 and ΔAccuracy were 0 across training fractions and strategies, as expected given the definition of the reference condition. This confirms that historical context is not sufficient to identify individuals on its own; its role is to re‐rank visually plausible candidates.

### 3.3 Sensitivity: Impact of Initialization

We further tested robustness to imperfect encounter membership by corrupting the initial confirmed identity set with probabilistic (binomial) noise, including (i) dropout (missing seeds), (ii) addition of decoy identities, and (iii) swaps (incorrect seeds), across corruption rates $p\in \left\{0,0.1,0.2,0.4,0.6\right\}$ and two training fractions (t=0.3,0.7). Performance was most robust to seed dropout: for the best‐performing strategy (Random Known, *n*=1), macro‐F1 remained essentially unchanged at t=0.7 even at high dropout (e.g., 0.741 at p=0 vs. 0.732 at p=0.6). Decoy additions caused a smooth reduction in gains (e.g., macro‐F1 at t=0.7 decreases from 0.741 to 0.697 as p increased to 0.6), consistent with dilution of the co‐occurrence signal. Seed swaps were the dominant failure mode, with macro‐F1 degrading most rapidly and approaching the ML‐only baseline at high corruption (e.g., decreasing from 0.741 to 0.673 at t=0.7), reinforcing that co‐occurrence improves identification when anchored to approximately correct encounter membership but can amplify errors under mis‐specification (Figure S4; Table S5).

### 3.4 Placebo Co‐Occurrence

#### 3.4.1 Placebo Log Lift Control (Fixed Weights)

To verify that improvements are attributable to meaningful encounter association structure rather than the mere presence of a contextual term, we constructed a placebo log lift matrix that preserves marginal label frequencies (and comparable support scale) while destroying true co‐occurrence relationships. We then re‐ran the single‐pass fusion update using fixed weights $\left(\alpha ,\beta ,\gamma \right)=\left(1.0,0.5,1.0\right)$ across headline initialization strategies. Under placebo context, fusion consistently degraded both accuracy and macro‐F1 relative to the ML‐only baseline across training regimes, whereas the same fixed‐weight update using real log lift yielded consistent gains for reliable initializations (Null Set, Top Unknown, Random Known). The macro‐F1 scores for training fraction t=0.7 using both the real and the placebo log lift data are shown in Table 2. The macro‐F1 scores for the remaining training fractions are presented visually in Figure S5.

**TABLE 2:** Macro‐F1 at training fraction t=0.7 for headline initialization strategies using real association log lift versus a placebo control in which log lift is computed from corrupted encounter co‐occurrence (see Section 2), with all other inference settings held constant.

Strategy	*n*	Macro F1_Real_	Macro F1_Placebo_	Real − Placebo
Baseline	—	0.651	—	—
Random Known	3	0.735	0.553	0.182
Random Known	2	0.734	0.557	0.177
Random Known	1	0.741	0.566	0.175
Top Unknown	1	0.735	0.57	0.165
Null Set	—	0.727	0.579	0.148
Random Unknown	—	0.669	0.563	0.106

*Note:* Values are mean across seeds (placebo and real evaluated on the same splits); “Real − Placebo” reports the absolute gain attributable to structured social context.

#### 3.4.2 Learning Fusion Weights With Placebo Log Lift

When allowing the weights to be optimized using the placebo log lift data instead of having them fixed, the fusion method downweights all co‐occurrence terms across all headline regimes and training fractions, resulting in the context weights being driven to 0 (β=0,γ=0). This results in no performance improvements for any metrics, as it is just a re‐creation of the ML‐only baseline. We therefore omit a redundant table and report this behavior descriptively.

### 3.5 Generality Diagnostics

To provide guidance beyond this case study, we quantified simple encounter‐log diagnostics that can be computed for any photo‐identification system: (i) the number of training encounters, (ii) the encounter size distribution, and (iii) the connectivity and repetition of the training co‐occurrence graph. In our data, the training‐history co‐occurrence graph matures rapidly as additional encounters are added: density increases from 0.212 at t=0.1 (mean degree 21.8) to 0.307 at t=0.2 (31.6) and 0.352 at t=0.3 (36.3), while the fraction of all possible dyads observed at least five times increases from 7.6% (t=0.1) to 11.8% (t=0.2) and 13.4% (t=0.3).

**FIGURE 7:**
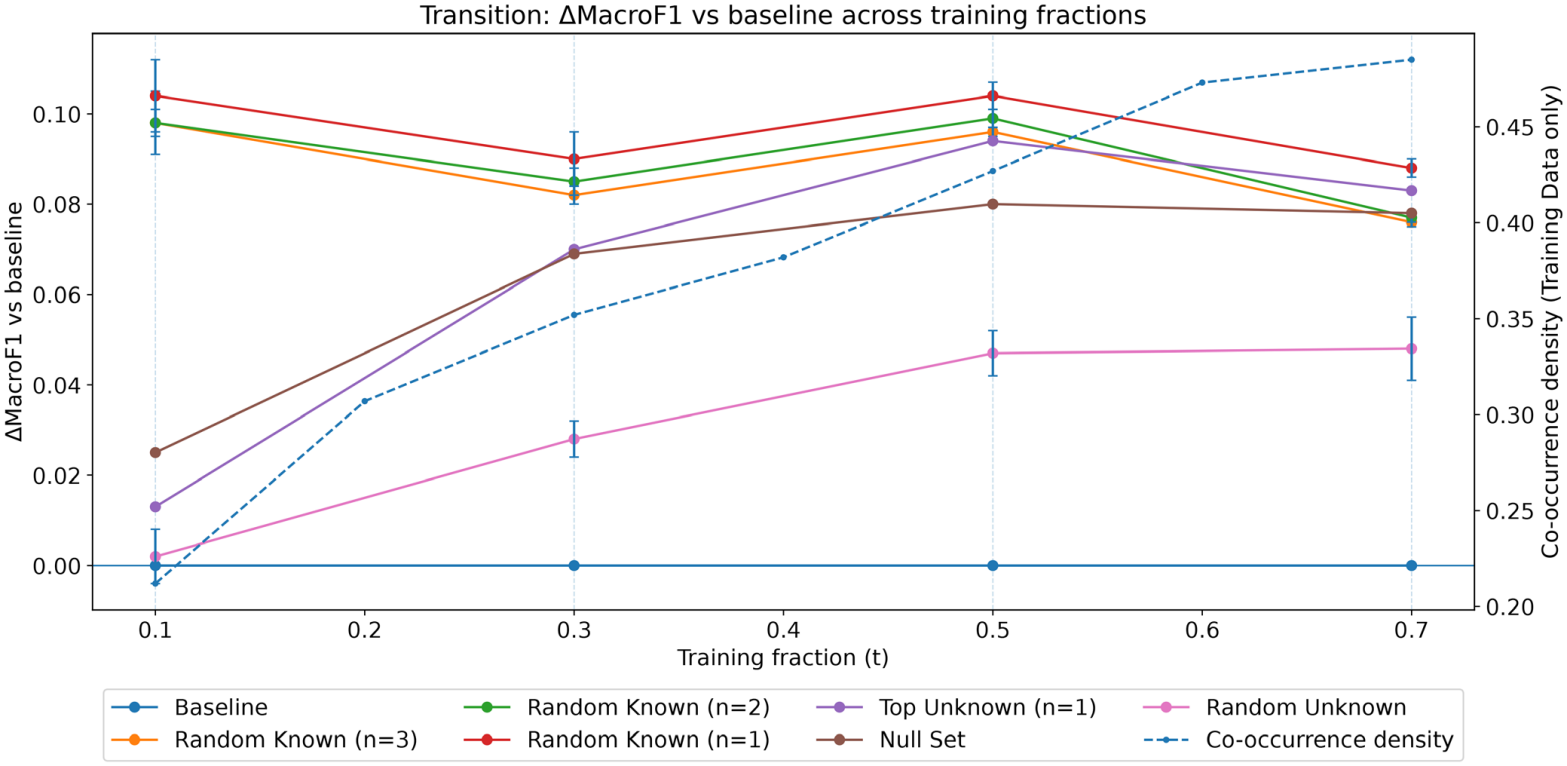
Mean macro‐F1 change relative to the ML‐only baseline (±SD for stochastic strategies) is shown for headline initialization strategies across training fractions $t\in \left\{0.1,0.3,0.5,0.7\right\}$. The dashed curve shows the density of the co‐occurrence graph computed from training encounters only. Together, the plots illustrate that gains broaden from primarily expert‐seeded settings at low training history (t=0.1) to multiple viable initialization regimes once co‐occurrence statistics stabilize (t≥0.3).

This maturation coincides with a qualitative shift in performance: at t=0.1, gains are largely confined to expert‐seeded initialization (Random Known), whereas from t≥0.3, multiple initialization regimes (Random Known, Null Set, Top Unknown) yield positive improvements over the ML‐only baseline. We emphasize these values as diagnostics from this study system rather than universal thresholds; nonetheless, they offer an interpretable checklist for when encounter‐conditioned co‐occurrence is likely to be informative. These diagnostics can be computed from any photo‐identification encounter log and therefore provide a simple way to assess whether co‐occurrence information is likely to be informative before applying the method. To make this transition concrete, Figure 7 plots macro‐F1 improvements across training regimes alongside the corresponding growth in co‐occurrence density.

Taken together, these generality diagnostics help explain when encounter‐conditioned co‐occurrence is likely to add value: it is most informative once repeated associations are sufficiently supported by training history and least reliable when encounter membership is uncertain or mis‐specified. Additional strategies, ablations, and full weight‐sweep results are provided in the Supporting Information.

## 4 Discussion

Across training fractions and evaluation regimes, a consistent pattern emerges: social co‐occurrence improves photo‐identification when it is anchored to reliable encounter membership information. This aligns with how association‐based inference is typically conducted in cetaceans and other social vertebrates: repeated co‐identifications yield non‐random, long‐lived association structure, and analysts routinely use that structure informally to constrain ambiguous matches (Farine and Whitehead 2015; Weiss et al. 2021; Whitehead 2008, 2009). In our experiments, providing even a small amount of expert input (Random‐*n* Known) yields the largest and most stable gains, consistent with the broader principle that a small amount of high‐precision human information can unlock disproportionate downstream benefit when propagated through structured data (Kellenberger et al. 2020; Norouzzadeh et al. 2018; Swanson et al. 2015). Fully automated seeding using confident model predictions (Top‐N Unknown) also improves performance, especially once sufficient training history exists to support both the classifier and the co‐occurrence statistics. This dependence on coverage and label quality echoes findings from large‐scale wildlife machine learning deployments that performance gains depend strongly on data coverage, label quality, and domain maturity (Christin et al. 2019; Tuia et al. 2022; Willi et al. 2019).

The sensitivity study highlights the importance of the initialization strategy and the source of the seed information. The method is most vulnerable to incorrect seeds: swaps rapidly erode gains because misleading encounter membership causes the context prior to reinforce the wrong candidates. It was, however, robust to seeds being dropped or to spurious identities being added, suggesting that missing or extra labels are less harmful as long as some truthful evidence remains. Operationally, this supports a precision‐first workflow: it is safer to confirm fewer identities than to confirm the wrong one.

The ablations reinforce a simple takeaway: historical context is not a substitute for visual evidence. Priors and co‐occurrence alone underperform the ML‐only baseline across training fractions, even under oracle encounter membership, underscoring that the fused model's gains come from using social structure to *re‐rank* plausible candidates, not from context independently identifying individuals in the absence of visual support. This finding is also consistent with long‐standing results in recognition systems: contextual cues improve decisions primarily by redistributing probability mass among plausible hypotheses, rather than creating hypotheses when the observation model provides little support (Galleguillos and Belongie 2010; Torralba 2003). Practically, this means co‐occurrence is most valuable as a lightweight post‐processing layer that redistributes probability mass among visually plausible identities, provided the encounter is seeded with at least some trustworthy membership information. The placebo control confirms that gains are driven by meaningful association structure: when co‐occurrence is corrupted but marginal frequencies are preserved, fixed‐weight fusion degrades performance relative to the ML‐only baseline (Table 2; Figure S5). When weights are learned under the placebo, the optimizer drives the co‐occurrence terms to zero, reverting to the baseline solution, indicating the weight‐selection procedure does not exploit artifacts in the fusion objective.

Weight selection shows that these gains are not dependent on a single fragile parameter setting. Across training fractions, the same small set of weight configurations repeatedly emerges as near‐optimal, and the staged optimization provides comparable or improved solutions relative to grid search for several strategies. This stability is consistent with classic results on combining probabilistic evidence sources: once features are moderately informative, a small family of fusion rules tends to perform robustly across regimes (Hinton 2002; Kittler et al. 1998). We select weights lexicographically (maximize macro‐F1; break ties by log loss, then Brier), so improvements reflect both discrimination and (when performance is otherwise indistinguishable) better‐calibrated probability estimates. This multi‐objective nature is often seen in probabilistic decision systems in ecology, where both ranking quality and probabilistic reliability matter for downstream decision‐making and workload triage. Proper scoring rules and calibration metrics (e.g., log loss, Brier score) provide principled ways to quantify that reliability (Brier 1950; Gneiting and Raftery 2007). Because the fusion formulation is fully differentiable, future work could also estimate these weights directly through gradient‐based optimization within a training pipeline rather than through validation‐based search.

Methodologically, this work extends a separate tradition of contextual or re‐ranking models in computer vision while introducing, to our knowledge, the first encounter‐conditioned fusion framework for wildlife identification that exploits real social structure. Our contribution is therefore distinct from prior ecological applications that adjust predictions heuristically or rely solely on temporal proximity. By representing co‐occurrence as a reusable log lift matrix derived from training encounters, we provide an interpretable, deployable mechanism that can be integrated into existing pipelines without architectural change. The black‐box nature of this approach is an important practical consideration given the rapid evolution of wildlife identification models and diversity of real‐world pipelines (Barnhill et al. 2025; Bergler et al. 2021; Patton et al. 2023; Tuia et al. 2022; Weinstein 2018). More broadly, this approach bridges two mature lines of ecological practice: (i) individual‐based inference built on photo‐identification and (ii) association‐based analysis of animal societies (Bigg et al. 1990; Hammond et al. 1990; Weiss et al. 2021; Whitehead 2008), by providing a transparent probabilistic mechanism for incorporating encounter structure directly into automated identification. Although we demonstrate the method using softmax classifier outputs, any identity scoring function that can be normalized across candidate identities (e.g., similarity scores from metric learning systems) could serve as the image evidence term.

From an operational perspective, the results suggest a practical semi‐automated workflow in which a small amount of reliable encounter‐level information can be propagated across images to improve identification accuracy. This is consistent with how human expertise is typically deployed in large archives: prioritizing high‐confidence anchors and using them to accelerate the remainder of the matching process. It also mirrors broader “human‐in‐the‐loop” patterns in ecological machine learning where interactive tooling can reduce annotation time while retaining expert oversight (Kellenberger et al. 2020; Norouzzadeh et al. 2018; Swanson et al. 2015). Importantly, our results also delineate a clear failure mode: when initialization is untrusted, context can reinforce incorrect labels. This motivates interface designs that emphasize precision‐first confirmation (few, reliable seeds) and make uncertainty visible, rather than maximizing automated propagation (Christin et al. 2019; Kellenberger et al. 2020).

Future work includes (i) evaluating the time savings and labeling quality of an interactive human‐in‐the‐loop interface, building on existing photo‐identification systems that couple model assistance with efficient expert correction (Kellenberger et al. 2020; Tuia et al. 2022), (ii) improving weight selection (e.g., Bayesian optimization or population‐based search) while retaining leakage‐safe validation, as automated hyperparameter tuning is often more sample‐efficient than exhaustive search in multi‐objective settings (Bergstra and Bengio 2012; Jaderberg et al. 2017; Snoek et al. 2012), and (iii) integrating encounter‐level structure more directly into training (e.g., joint learning with graph‐structured context) rather than post hoc fusion, for example by learning with graph‐structured context or attention over encounter membership. Modern graph neural network methods provide a natural template for this direction (Kipf and Welling 2016; Veličković et al. 2018), while ecological best practices for network construction and interpretation clarify what such models should preserve (Farine and Whitehead 2015; Whitehead 2008). Finally, the same approach should transfer to other long‐lived, socially structured species where repeated co‐occurrence is common, provided encounter definitions are reliable and sufficient historical data exist to estimate stable associations. This includes other toothed whales and delphinids where association patterns are routinely quantified (Augusto et al. 2017; Towers et al. 2025; Weiss et al. 2021), as well as terrestrial systems where group sightings or burst sampling are common (e.g., primates, elephants, ungulates, and camera‐trap sequences). In such settings, context‐assisted re‐ranking may be especially valuable when visual distinctiveness is limited or when images are heterogeneous, provided evaluation respects temporal/spatial structure to avoid optimistic leakage (Roberts et al. 2017; Willi et al. 2019). Social‐network analysis can provide both a general methodological framework for quantifying association structure (Croft et al. 2008) and, across taxa, a basis for testing how social structure covaries with individual traits and outcomes (Croft et al. 2009; Ellis et al. 2017).

### 4.1 Practical Implications (Recommended Workflow)

- Define the encounter unit (what constitutes a single co‐occurrence event) and ensure membership is recorded consistently.
- Build an initial labeled catalog of individual–image pairs to train a baseline classifier.
  ○ Rule of thumb: 30–50 labeled images per individual (high‐quality, diverse viewpoints) to reach a useful baseline classifier
  ○ For robust generalization and fewer long‐tail failures: 100–300 images per individual where feasible; more if individuals are visually similar or image quality is variable.
- Estimate historical context from training data only: (i) individual priors and (ii) the co‐occurrence matrix.
- Operational labeling loop (per new encounter):
  ○ Run the classifier on all detections.
  ○ Initialize the encounter with minimal high‐confidence information (e.g., 1–3 expert‐confirmed individuals), or use the most confident model predictions in fully automated settings.
  ○ Fuse classifier posteriors with co‐occurrence scores to re‐rank candidates across the encounter.
  ○ Auto‐label conservatively (only promote predictions above a strict threshold) and route ambiguous cases to a specialist.
- Iterate: periodically retrain using newly confirmed labels, updating priors and co‐occurrence from the expanded labeled set.

When it helps most: co‐occurrence contributes most when (i) encounter membership is reliable and (ii) the classifier is not yet strong enough to resolve ambiguous individuals consistently. As the classifier improves, the relative gains from co‐occurrence tend to diminish, though context can still provide operational benefits for rare individuals or low‐quality images.

### 4.2 Timing Considerations

We report runtimes from a consumer laptop (Lenovo X1 Carbon; Intel Core i7‐1255U; 16 GB RAM; CPU‐only). Grid‐based weight selection is the dominant computational cost and is slowest in the smallest training regime: at t=0.1, selection averaged ~21 h per run (p95 ~ 44 h), whereas at t=0.7 selection averaged ~13 h (p95 ~ 24 h). In contrast, inference is lightweight: encounter‐level evaluation remains < 0.13 s per encounter (typically ~0.08–0.10 s, p95 ~ 0.20–0.27 s) with minimal variation across initialization modes. Fusion adds only a small per‐image overhead (~0.11–0.17 ms), so total runtime is dominated by the model forward pass and remains stable across strategies.

## Author Contributions

**Alexander Barnhill:** conceptualization (lead), formal analysis (lead), investigation (lead), methodology (lead), software (lead), visualization (lead), writing – original draft (lead). **Jared R. Towers:** data curation (supporting), investigation (supporting), resources (supporting), writing – review and editing (supporting). **Gary J. Sutton:** data curation (supporting), investigation (supporting), writing – review and editing (supporting). **Tasli J. H. Shaw:** data curation (supporting), investigation (supporting), writing – review and editing (supporting). **Thomas Doniol‐Valcroze:** resources (supporting), supervision (supporting), writing – review and editing (supporting). **Elmar Nöth:** supervision (supporting), writing – review and editing (supporting). **Andreas Maier:** supervision (supporting), writing – review and editing (supporting). **Christian Bergler:** supervision (supporting), writing – review and editing (supporting).

## Disclosure

Statement on Inclusion: This study uses a long‐term photo‐identification archive from coastal British Columbia, Canada. The authorship team includes Canada‐based scientists and practitioners from the organizations involved in data collection and population monitoring, who contributed to study design, interpretation, and manuscript revision. Results and derived outputs are being shared back with contributing partners to support ongoing photo‐identification and monitoring activities, subject to contributor permissions and data‐use agreements.

## Conflicts of Interest

The authors declare no conflicts of interest.

## Supporting information


**Data S1:** ece373552‐sup‐0001‐DataS1.pdf.

## Data Availability

Code and derived artifacts required to reproduce all analyses are archived on Dryad (DOI: https://doi.org/10.5061/dryad.pzgmsbd2n), including encounter split files (with relative chronological order only), per‐image classifier probability outputs for evaluation splits, global sighting priors, and co‐occurrence matrices. Encounter metadata (dates, locations, photographer information) and individual identifiers have been anonymized using non‐reversible pseudonyms. This anonymization preserves the relational and temporal structure of the data required for the analysis while removing identifying information about contributors and locations. The original photographs are owned by multiple contributing organizations and photographers and cannot be redistributed due to third‐party ownership and licensing restrictions. Integration of this workflow into broader wildlife re‐identification tool ecosystems is a useful direction for future work. Data Sources: Photographic encounter data were compiled from long‐term photo‐identification efforts by Bay Cetology and collaborators, with contributions from Fisheries and Oceans Canada (Pacific Biological Station) and Ocean Wise, and additional contributing organizations and photographers as permitted under data‐use agreements.
