# Supplementary material for: Leveraging Co‐Occurrence to Improve Deep Learning Photo‐Identification in Social Animals
Source: Ecol Evol. 2026 Apr 21;16(4):e73552. doi: 10.1002/ece3.73552 (PMC13099174; doi:10.1002/ece3.73552)
Supplement: Supplementary file 1 — Data S1: ece373552‐sup‐0001‐DataS1.pdf. [file ECE3-16-e73552-s001.pdf]

Leveraging Co-Occurrence to Improve Deep Learning Photo-Identification in Social Animals : Supporting Information

This Supporting Information contains: (S1) full definitions of initialization strategies; (S2) ablation definitions; (S3) weight search (grid + optimization) procedures; (S4) additional figures; (S5) full tables of results.

Appendix S1. Initialization Strategies

For all strategies where sampling is performed, sampling is without replacement; duplicates are ignored until the target size is reached or the candidate pool is exhausted.

S1.1 Headline Strategies (reported in main text)

1. **Random n Known.** Uniformly sample n unique ground-truth identities present in the encounter, without specifying which images they correspond to. This models a human-in-the-loop workflow where an analyst can confirm a small number of individuals are present.

2. **Top n Unknown.** Select the n unique identities corresponding to the highest-confidence baseline predictions in the encounter (sorted by $\max_y p(y \mid x)$ per image). This is an “easy-first” self-seeding strategy.

3. **Null Set.** Initialize with no confirmed identities: $S_0 = \emptyset$. This tests whether the procedure can bootstrap context purely through the subsequent encounter update.

4. **Random Unknown.** Uniformly sample one unique identity from the set of baseline predicted labels appearing anywhere in the encounter (i.e., the set of per-image argmax predictions).

S1.2 Additional Strategies

5. **Random n Unknown with Frequency Weighting.** Sample n unique identities from the set of baseline predicted labels, weighted by within-encounter frequency (how often each label is predicted as argmax across the encounter). We highlight one frequency transform, namely taking the square root of the count as the weight. Other transformations such as the raw count or a log transformation are left for future work. This transform controls how strongly repeated predictions are favored.

6. **Highest n Entropy.** Identify the n unique predicted labels associated with the highest-entropy baseline posteriors in the encounter (i.e., the most uncertain images), returning each selected image’s argmax label. This probes whether “hard” images provide useful seeds. Entropy is defined as $H(p) = -\sum_y p(y) \log p(y)$, for all entropy-based methods.

7. **Lowest n Entropy.** Identify the n unique predicted labels associated with the lowest-entropy baseline posteriors (the most confident images), returning each selected image's argmax label. This is similar in intent to Top Unknown, but ranks by entropy rather than peak probability.

8. **Random Known n with Frequency Weighting.** As above, but sample encounter ground-truth identities weighted by their within-encounter frequency (i.e., number of images in which each individual appears), optionally transformed. This reflects that frequently photographed individuals may be easier to confirm.

9. **ORACLE — All Known.** Initialize with the full set of unique ground-truth identities present in the encounter (membership known perfectly, still without image-level assignment). This provides an upper bound on what the context term can contribute if encounter membership were known.

10. **Top Prior n.** Initialize with the n identities with highest global prior probability $\pi(y)$.

11. **Random Prior n.** Uniformly sample n identities from the global label universe (excluding any already in S_0, if applicable).

12. **Random Weighted Prior n.** Sample n identities from the global label universe weighted by $\pi(y)$, reflecting that commonly sighted individuals are more plausible encounter members.

Appendix S2. Ablation Definitions

1. **ML-only ($\alpha = 1, \beta = 0, \gamma = 0$):** Predictions depend only on the ML classifier.

2. **Priors-only ($\alpha = 0, \beta = 1, \gamma = 0$):** Predictions depend only on the prior.

3. **Priors + log lift, ML guidance only (no ML in fusion) ($\alpha = 0, \beta > 0, \gamma > 0$):** Fusion uses priors and co-occurrence, while the ML model is used only to initialize S_0 probabilities (e.g., Top Unknown).

4. **Log lift only, ML guidance only ($\alpha = 0, \beta = 0, \gamma > 0$):** Fusion uses co-occurrence alone (with the empty-set case falling back to a uniform distribution, and ties broken deterministically by argmax); ML is used only for guidance (initialization and/or ordering).

5. **Priors + log lift with no ML guidance ($\alpha = 0, \beta > 0, \gamma > 0$):** Fusion is based on priors and co-occurrence, but with initialization based on priors (e.g., Top Prior) instead of ML predictions, with the order of predictions being randomized instead of being sorted by probability. This isolates a fully non-ML procedure that relies solely on training-history statistics.

6. **Oracle initialization (upper bound for non-ML guidance):** Same as (5) but initialized with the set of ground-truth identities present in the encounter (without assigning identities to images). This quantifies the potential gain if encounter membership were known perfectly.

Appendix S3. Weight Search Procedures

Model-Selection Criterion (Lexicographic). Weights are selected on the validation split using a lexicographic criterion. We first identify the highest mean validation macro-F1. We then form a "near-optimal" set consisting of all configurations whose mean macro-F1 lies within one

standard deviation of the best value, where the standard deviation is estimated across five random restarts (different random initializations of the coordinate-wise search). Among configurations in this near-optimal set, we select the one with lowest mean log loss; remaining ties are broken by lowest mean Brier score. This rule prioritizes discrimination (macro-F1) while favoring better-calibrated probabilistic predictions when performance is otherwise indistinguishable.

Grid Search for Optimal Weights. As a baseline, we perform a staged grid search on the validation split. We first select the prior-image weights while fixing co-occurrence weight to zero, evaluating a discrete grid ($\alpha, \beta \in \{0.5, 1, 1.5, 2\}$). For each training fraction and initialization strategy, we retain the best setting under the lexicographic criterion above.

Given the selected prior-image weights, we then sweep the co-occurrence weight over $\gamma \in \{0.5, 1, 1.5, 2\}$ on the validation split and retain the best value under the same lexicographic criterion. This staging isolates the effect of adding the co-occurrence term from the prior-image fusion.

Appendix S4. Additional Figures

This section provides the figures for both the grid search (Figure S1) and staged optimization (Figure S2) results in terms of mean Macro-F1 scores for each initialization strategy and training split. Furthermore we show the Pareto frontier results for all training fractions to demonstrate the calibration–discrimination trade-off for both the grid search and the optimization-selected weights (Figure S3). Sensitivity results showing the effect of seed corruption are shown in Figure S4. Results showing the effect of corrupted associative data are shown in Figure S5.

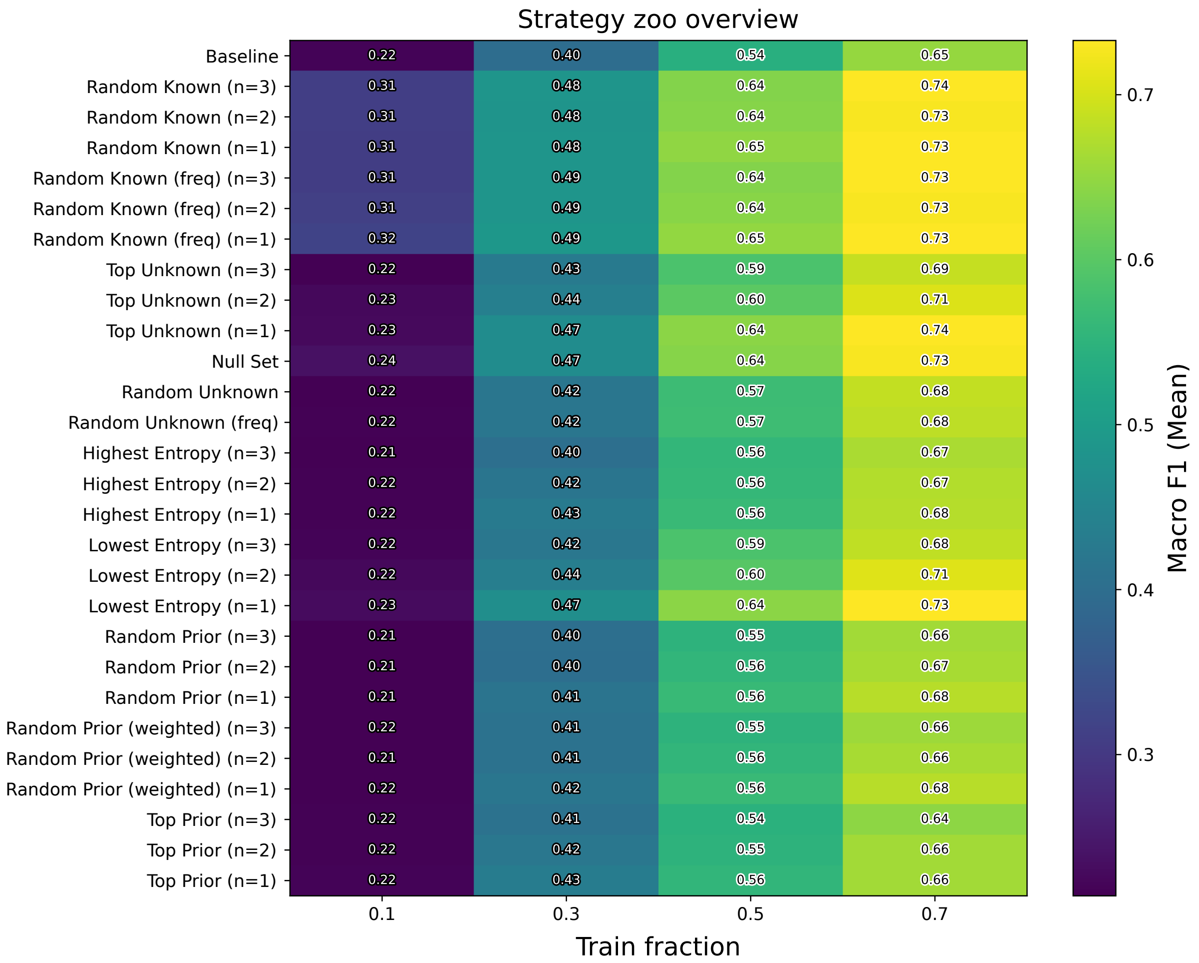

Supplementary Figure S1. Strategy "zoo" heatmap of mean test Macro F1 across training fractions for all evaluated initialization strategies using the staged grid search (baseline shown for reference). For each strategy and training fraction, we plot the best result from the selected fusion-weight configuration (α, β, γ). Values are mean across seeds for stochastic strategies; deterministic strategies are single runs.

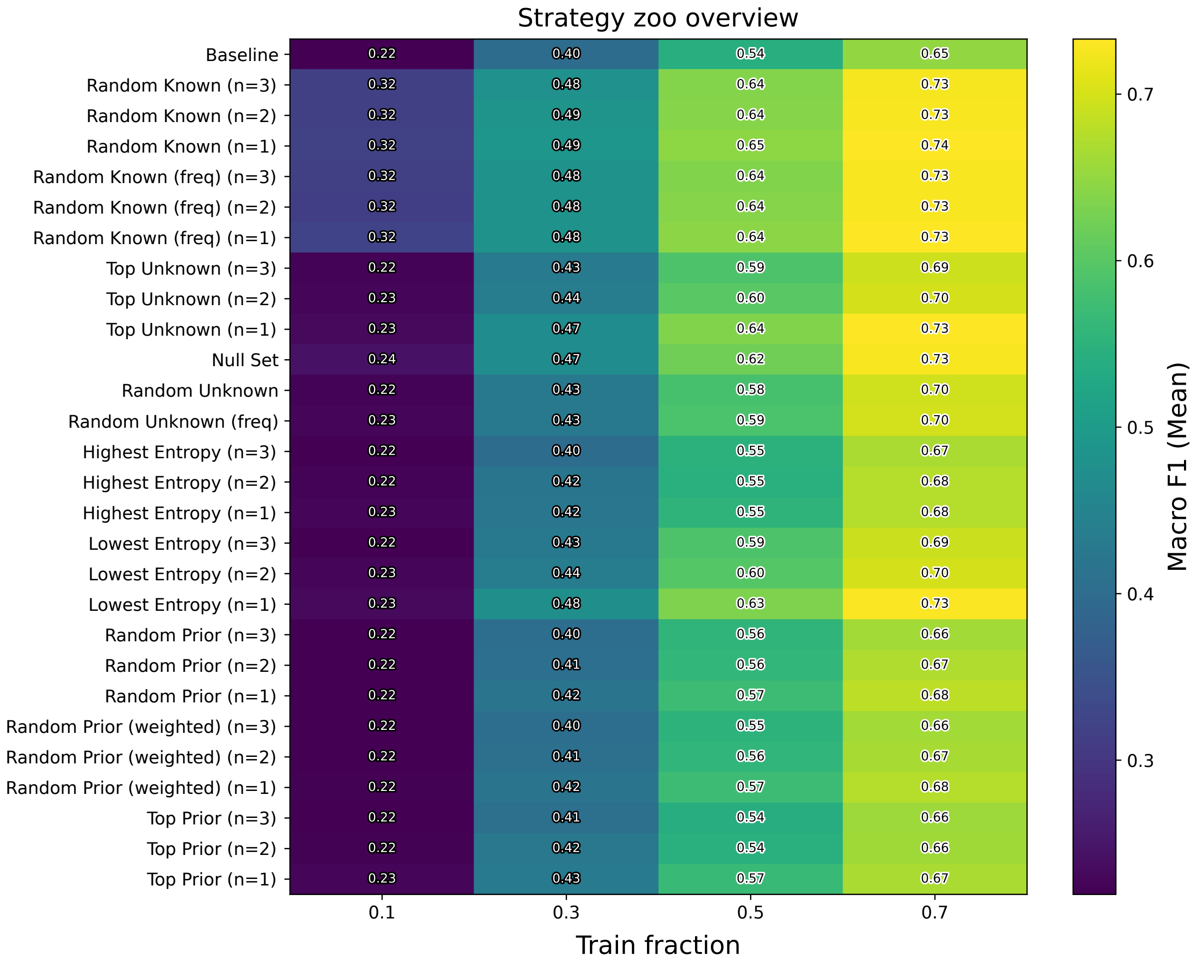

Supplementary Figure S2. Strategy "zoo" heatmap of mean test Macro F1 across training fractions for all evaluated initialization strategies using the coordinate-wise (staged) optimization procedure (baseline shown for reference). For each strategy and training fraction, we plot the selected fusion-weight configuration (α, β, γ) from the search using the staged tie-break in Methods. Values are mean across seeds for stochastic strategies; deterministic strategies are single runs.

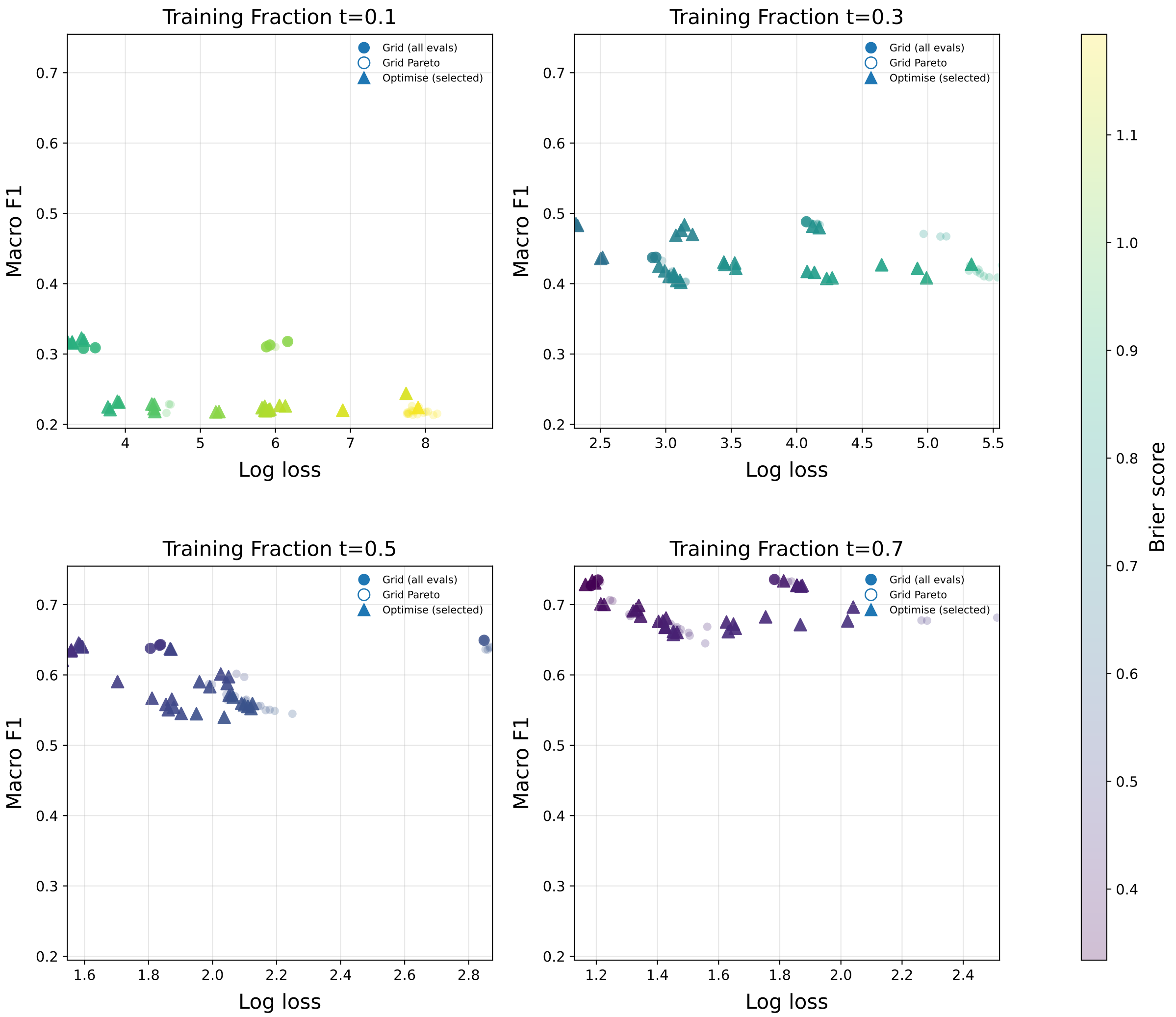

Supplementary Figure S3. Pareto frontier search results across all training fractions. Circles show grid-evaluated weight combinations (faint) with Pareto-optimal grid solutions overlaid; triangles indicate the optimization-selected weights. Point color encodes Brier score using a shared color scale across panels, highlighting the calibration–discrimination trade-off. We select weights lexicographically (maximize macro-F1; break ties by log loss then Brier). We additionally plot Pareto frontiers over these metrics to visualize calibration–discrimination trade-offs. Y-axis limits are shared across panels to aid comparison, while x-axis limits are set per panel to preserve resolution where log loss ranges differ. Summary statistics for each training fraction (number of evaluations, Pareto set size, and Pareto hit-rate of selected solutions) are reported in Supplementary Table S3.

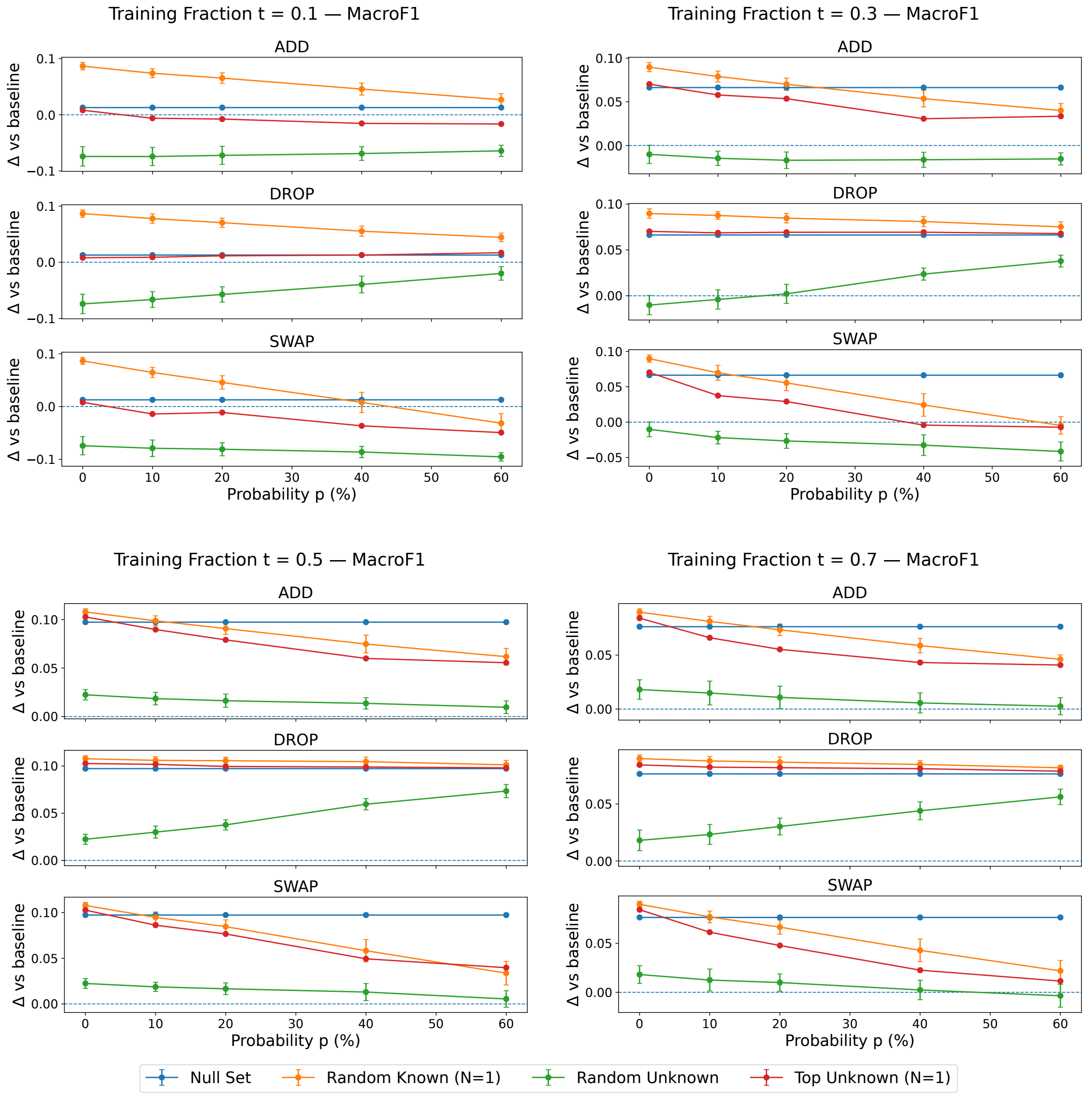

Supplementary Figure S4. Results for the sensitivity experiments investigating the impact of set initialization on performance. We report the absolute change in macro-F1 (ΔF1 vs baseline, SD over 10 seeds) with respect to the ML-only baseline for each of the three initialization corruption methods for all training fractions across each of the four headline initialization strategies. Corruption is applied stochastically with probability ε under three modes: drop (remove each seed label with probability ε), swap (replace each seed label with probability ε by a label not already present), and add ($\varepsilon|S_0|$ decoy labels). Negative values indicate degradation. The null set initialization strategy is included for reference.

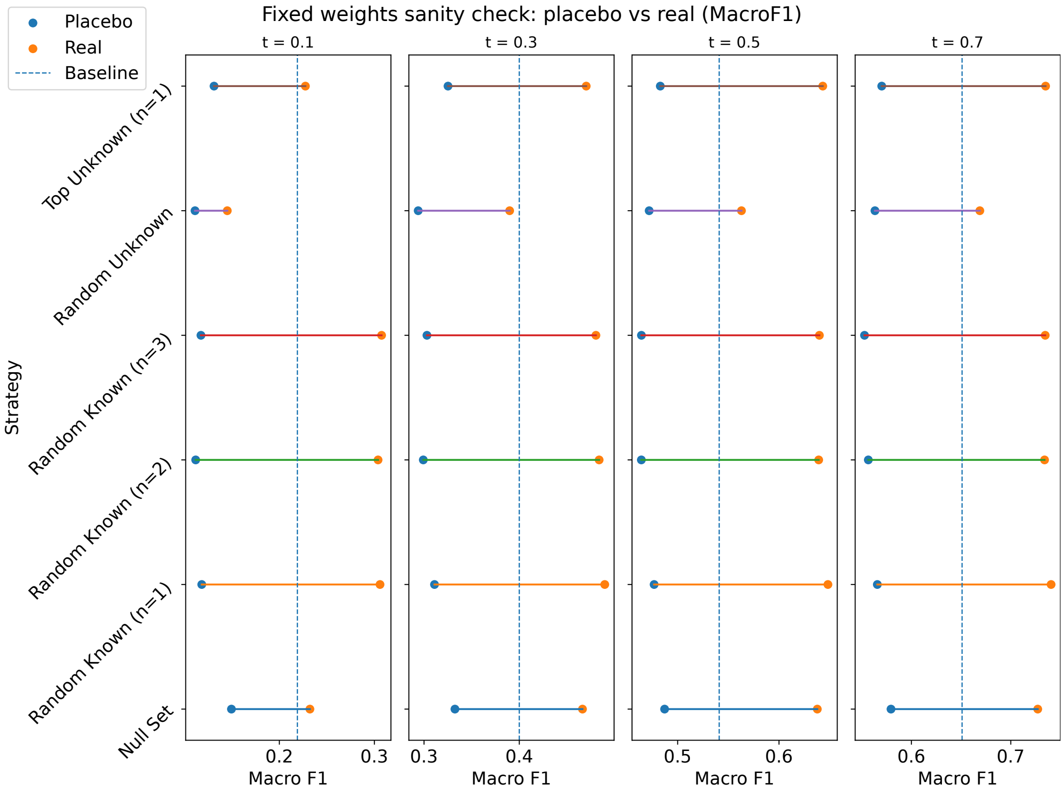

Supplementary Figure S5. The differences in mean Macro F1 scores across the headline initialization strategies and all training fractions when using both the real associative data for the creation of the log lift matrix and when using placebo data generated using the original data and then shuffling it to remove any associative information but retaining marginal label frequencies. The ML-only baseline is plotted as a vertical dotted line to provide context to the changing results.

Appendix S5. Full Results Tables

This section includes the summary tables for the grid search (Table S1) and optimization-selected weights (Table S2). These results include the selected weights for every strategy and training fraction, as well as the resulting mean macro-F1 scores and accuracy. Table S3 shows the optimization details for the fusion weight selection. Table S4 summarizes the ablation studies and their performance with respect to the ML-classifier-only baseline. Table S5 presents the results from the sensitivity study which investigates the impact of seed corruption at the start of the fusion process.

Supplementary Table S1. Results from the grid search across all training fractions for optimal weights for the fusion process. Alongside training fraction (t) and Strategy with accompanying n where applicable, we report the selected weights (α, β, γ), the mean across 10 seeds with ($\pm$ SD) for Macro-F1, Accuracy, Log Loss, Brier Score, and relative Accuracy Error Reduction Rate (rAER).

t	Strategy	n	α	β	γ	Macro F1	Accuracy	Log Loss	Brier	rAER
0.1	Baseline	—	—	—	—	0.219	0.263	—	—	0
0.1	Random Known	3	2	0.5	2	0.310 (0.002)	0.360 (0.003)	5.880 (0.061)	1.047 (0.004)	0.132 (0.004)
0.1	Random Known	2	2	0.5	2	0.310 (0.004)	0.360 (0.005)	6.000 (0.067)	1.048 (0.007)	0.131 (0.007)
0.1	Random Known	1	1	0.5	1	0.309 (0.012)	0.355 (0.012)	3.599 (0.055)	0.897 (0.015)	0.124 (0.016)
0.1	Random Known (freq)	3	1	0.5	1	0.308 (0.004)	0.355 (0.004)	3.442 (0.033)	0.895 (0.004)	0.124 (0.006)
0.1	Random Known (freq)	2	2	0.5	2	0.313 (0.003)	0.364 (0.003)	5.927 (0.056)	1.044 (0.005)	0.136 (0.004)
0.1	Random Known (freq)	1	2	0.5	2	0.318 (0.007)	0.366 (0.005)	6.163 (0.084)	1.040 (0.006)	0.139 (0.007)
0.1	Top Unknown	3	1	0.5	0.5	0.216	0.262	4.548	0.974	−0.001
0.1	Top Unknown	2	2	0.5	1	0.226	0.270	7.819	1.173	0.009
0.1	Top Unknown	1	1	0.5	0.5	0.228	0.283	4.603	0.972	0.026
0.1	Null Set	—	2	0.5	2	0.238	0.292	9.829	1.188	0.039
0.1	Random Unknown	—	2	0.5	1	0.216 (0.003)	0.262 (0.003)	7.972 (0.125)	1.187 (0.005)	−0.002 (0.004)
0.1	Random Unknown (freq)	—	2	0.5	1	0.218 (0.004)	0.263 (0.004)	8.003 (0.128)	1.186 (0.006)	−0.001 (0.005)
0.1	Highest Entropy	3	2	0.5	0.5	0.215	0.261	7.753	1.181	−0.003
0.1	Highest Entropy	2	2	0.5	1	0.218	0.262	7.857	1.182	−0.002
0.1	Highest Entropy	1	2	0.5	1	0.218	0.263	8.033	1.183	0
0.1	Lowest Entropy	3	2	0.5	1	0.218	0.261	7.830	1.185	−0.003
0.1	Lowest Entropy	2	2	0.5	1	0.225	0.267	7.910	1.181	0.005
0.1	Lowest Entropy	1	1	0.5	0.5	0.229	0.282	4.581	0.972	0.026
0.1	Random Prior	3	2	0.5	1	0.213 (0.003)	0.258 (0.002)	7.832 (0.023)	1.185 (0.004)	−0.007 (0.003)
0.1	Random Prior	2	2	0.5	1	0.214 (0.004)	0.258 (0.003)	7.893 (0.045)	1.187 (0.006)	−0.007 (0.005)
0.1	Random Prior	1	2	0.5	1	0.213 (0.004)	0.257 (0.003)	8.105 (0.097)	1.193 (0.007)	−0.008 (0.004)
0.1	Random Prior (weighted)	3	2	0.5	0.5	0.215 (0.002)	0.260 (0.001)	7.770 (0.012)	1.181 (0.002)	−0.005 (0.002)
0.1	Random Prior (weighted)	2	2	0.5	0.5	0.215 (0.002)	0.260 (0.001)	7.775 (0.012)	1.181 (0.002)	−0.004 (0.002)
0.1	Random Prior (weighted)	1	2	0.5	1	0.215 (0.005)	0.260 (0.003)	8.156 (0.098)	1.192 (0.007)	−0.004 (0.005)
0.1	Top Prior	3	2	0.5	0.5	0.217	0.263	7.769	1.178	−0.001
0.1	Top Prior	2	2	0.5	1	0.217	0.262	7.756	1.181	−0.002
0.1	Top Prior	1	2	0.5	1	0.220	0.265	7.812	1.180	0.002
0.3	Baseline	—	—	—	—	0.400	0.463	—	—	0
0.3	Random Known	3	2	0.5	2	0.484 (0.003)	0.544 (0.002)	4.130 (0.030)	0.766 (0.003)	0.151 (0.004)
0.3	Random Known	2	2	0.5	2	0.484 (0.004)	0.542 (0.005)	4.176 (0.069)	0.768 (0.007)	0.147 (0.009)
0.3	Random Known	1	2	0.5	2	0.485 (0.004)	0.542 (0.003)	4.161 (0.095)	0.764 (0.004)	0.148 (0.005)
0.3	Random Known (freq)	3	2	0.5	2	0.485 (0.002)	0.544 (0.002)	4.120 (0.029)	0.767 (0.002)	0.151 (0.003)
0.3	Random Known (freq)	2	2	0.5	2	0.485 (0.003)	0.543 (0.002)	4.157 (0.055)	0.768 (0.004)	0.148 (0.004)
0.3	Random Known (freq)	1	2	0.5	2	0.488 (0.003)	0.544 (0.003)	4.073 (0.072)	0.762 (0.006)	0.151 (0.006)
0.3	Top Unknown	3	2	0.5	2	0.426	0.481	5.566	0.862	0.033
0.3	Top Unknown	2	1	0.5	0.5	0.438	0.499	2.925	0.701	0.067
0.3	Top Unknown	1	2	0.5	2	0.467	0.523	5.142	0.800	0.111
0.3	Null Set	—	2	0.5	2	0.467	0.522	5.096	0.800	0.110

continued on next page

Supplementary Table S1 — continued from previous page

t	Strategy	n	α	β	γ	Macro F1	Accuracy	Log Loss	Brier	rAER
0.3	Random Unknown	—	2	0.5	1	0.419 (0.006)	0.481 (0.005)	5.315 (0.067)	0.857 (0.008)	0.032 (0.010)
0.3	Random Unknown (freq)	—	1	0.5	0.5	0.417 (0.008)	0.478 (0.006)	3.047 (0.033)	0.726 (0.006)	0.027 (0.012)
0.3	Highest Entropy	3	1	0.5	0.5	0.404	0.469	3.107	0.735	0.010
0.3	Highest Entropy	2	1	0.5	0.5	0.416	0.477	3.046	0.724	0.025
0.3	Highest Entropy	1	2	0.5	1	0.426	0.485	5.323	0.854	0.041
0.3	Lowest Entropy	3	1	0.5	0.5	0.420	0.485	2.984	0.716	0.041
0.3	Lowest Entropy	2	1	0.5	0.5	0.437	0.499	2.899	0.702	0.066
0.3	Lowest Entropy	1	2	0.5	2	0.471	0.525	4.968	0.796	0.114
0.3	Random Prior	3	1	0.5	0.5	0.403 (0.003)	0.467 (0.004)	3.153 (0.019)	0.736 (0.005)	0.008 (0.007)
0.3	Random Prior	2	1	0.5	0.5	0.402 (0.006)	0.467 (0.007)	3.150 (0.029)	0.736 (0.009)	0.006 (0.012)
0.3	Random Prior	1	2	0.5	1	0.415 (0.003)	0.477 (0.003)	5.399 (0.051)	0.861 (0.006)	0.025 (0.006)
0.3	Random Prior (weighted)	3	2	0.5	1	0.409 (0.002)	0.473 (0.002)	5.469 (0.032)	0.869 (0.003)	0.018 (0.004)
0.3	Random Prior (weighted)	2	2	0.5	1	0.410 (0.002)	0.475 (0.002)	5.430 (0.030)	0.865 (0.003)	0.021 (0.003)
0.3	Random Prior (weighted)	1	2	0.5	1	0.417 (0.005)	0.481 (0.005)	5.374 (0.060)	0.855 (0.009)	0.033 (0.010)
0.3	Top Prior	3	2	0.5	2	0.409	0.470	5.534	0.872	0.013
0.3	Top Prior	2	2	0.5	2	0.420	0.477	5.387	0.864	0.025
0.3	Top Prior	1	1	0.5	0.5	0.433	0.484	2.973	0.720	0.039
0.5	Baseline	—	—	—	—	0.541	0.587	—	—	0
0.5	Random Known	3	2	0.5	2	0.636 (0.002)	0.666 (0.002)	2.859 (0.019)	0.561 (0.002)	0.192 (0.004)
0.5	Random Known	2	2	0.5	2	0.638 (0.003)	0.667 (0.002)	2.865 (0.028)	0.561 (0.003)	0.193 (0.005)
0.5	Random Known	1	2	0.5	2	0.646 (0.004)	0.672 (0.003)	2.884 (0.046)	0.552 (0.006)	0.206 (0.008)
0.5	Random Known (freq)	3	2	0.5	2	0.636 (0.003)	0.666 (0.002)	2.852 (0.018)	0.561 (0.002)	0.192 (0.005)
0.5	Random Known (freq)	2	2	0.5	2	0.640 (0.002)	0.667 (0.002)	2.867 (0.021)	0.561 (0.002)	0.193 (0.004)
0.5	Random Known (freq)	1	2	0.5	2	0.649 (0.004)	0.674 (0.003)	2.848 (0.032)	0.548 (0.006)	0.213 (0.008)
0.5	Top Unknown	3	1	0.5	0.5	0.587	0.624	1.989	0.531	0.089
0.5	Top Unknown	2	1	0.5	1	0.602	0.639	2.075	0.518	0.126
0.5	Top Unknown	1	1	0.5	1	0.643	0.671	1.838	0.475	0.204
0.5	Null Set	—	1	0.5	1	0.638	0.667	1.806	0.476	0.195
0.5	Random Unknown	—	1	0.5	0.5	0.570 (0.009)	0.611 (0.007)	2.070 (0.043)	0.543 (0.007)	0.058 (0.016)
0.5	Random Unknown (freq)	—	1	0.5	0.5	0.572 (0.009)	0.613 (0.007)	2.043 (0.049)	0.540 (0.008)	0.065 (0.018)
0.5	Highest Entropy	3	1	0.5	0.5	0.558	0.600	2.121	0.551	0.033
0.5	Highest Entropy	2	1	0.5	0.5	0.560	0.605	2.099	0.548	0.045
0.5	Highest Entropy	1	1	0.5	0.5	0.565	0.609	2.045	0.541	0.054
0.5	Lowest Entropy	3	1	0.5	0.5	0.586	0.622	1.998	0.532	0.086
0.5	Lowest Entropy	2	1	0.5	1	0.597	0.633	2.099	0.524	0.113
0.5	Lowest Entropy	1	1	0.5	1	0.642	0.671	1.835	0.475	0.203
0.5	Random Prior	3	1	0.5	0.5	0.550 (0.003)	0.594 (0.003)	2.166 (0.017)	0.561 (0.005)	0.017 (0.008)
0.5	Random Prior	2	1	0.5	0.5	0.556 (0.004)	0.600 (0.004)	2.142 (0.017)	0.557 (0.004)	0.033 (0.009)
0.5	Random Prior	1	1	0.5	0.5	0.564 (0.006)	0.607 (0.005)	2.102 (0.028)	0.549 (0.007)	0.048 (0.013)
0.5	Random Prior (weighted)	3	1	0.5	0.5	0.551 (0.003)	0.594 (0.003)	2.178 (0.015)	0.564 (0.004)	0.017 (0.007)
0.5	Random Prior (weighted)	2	1	0.5	0.5	0.556 (0.005)	0.599 (0.004)	2.150 (0.017)	0.558 (0.005)	0.030 (0.010)
0.5	Random Prior (weighted)	1	1	0.5	0.5	0.565 (0.003)	0.606 (0.003)	2.105 (0.018)	0.549 (0.006)	0.047 (0.006)
0.5	Top Prior	3	1	0.5	0.5	0.545	0.588	2.249	0.577	0.004
0.5	Top Prior	2	1	0.5	0.5	0.549	0.591	2.194	0.574	0.010
0.5	Top Prior	1	1	0.5	0.5	0.556	0.599	2.123	0.560	0.029
0.7	Baseline	—	—	—	—	0.651	0.682	—	—	0
0.7	Random Known	3	2	0.5	2	0.736 (0.001)	0.755 (0.001)	1.783 (0.009)	0.402 (0.001)	0.229 (0.002)
0.7	Random Known	2	2	2	2	0.728 (0.001)	0.748 (0.001)	1.869 (0.010)	0.412 (0.001)	0.206 (0.004)
0.7	Random Known	1	2	2	2	0.733 (0.002)	0.753 (0.002)	1.839 (0.033)	0.399 (0.004)	0.222 (0.006)
0.7	Random Known (freq)	3	2	1	2	0.732 (0.001)	0.752 (0.001)	1.801 (0.010)	0.405 (0.001)	0.220 (0.002)
0.7	Random Known (freq)	2	2	2	2	0.727 (0.001)	0.747 (0.001)	1.860 (0.014)	0.412 (0.002)	0.204 (0.003)

continued on next page

Supplementary Table S1 — continued from previous page

t	Strategy	n	α	β	γ	Macro F1	Accuracy	Log Loss	Brier	rAER
0.7	Random Known (freq)	1	2	2	2	0.733 (0.004)	0.752 (0.003)	1.828 (0.030)	0.400 (0.005)	0.219 (0.010)
0.7	Top Unknown	3	1	0.5	0.5	0.686	0.718	1.308	0.386	0.111
0.7	Top Unknown	2	1	0.5	0.5	0.705	0.733	1.253	0.368	0.158
0.7	Top Unknown	1	1	0.5	1	0.735	0.759	1.205	0.339	0.242
0.7	Null Set	—	1	0.5	1	0.727	0.756	1.184	0.341	0.232
0.7	Random Unknown	—	1	0.5	0.5	0.685 (0.004)	0.716 (0.005)	1.342 (0.034)	0.389 (0.006)	0.104 (0.015)
0.7	Random Unknown (freq)	—	2	0.5	2	0.682 (0.013)	0.711 (0.011)	2.511 (0.081)	0.473 (0.018)	0.090 (0.035)
0.7	Highest Entropy	3	1	0.5	0.5	0.668	0.699	1.464	0.414	0.054
0.7	Highest Entropy	2	1	0.5	0.5	0.673	0.703	1.443	0.410	0.066
0.7	Highest Entropy	1	1	0.5	0.5	0.676	0.710	1.403	0.399	0.088
0.7	Lowest Entropy	3	1	0.5	0.5	0.684	0.716	1.311	0.388	0.106
0.7	Lowest Entropy	2	1	0.5	0.5	0.707	0.733	1.245	0.368	0.160
0.7	Lowest Entropy	1	1	0.5	1	0.732	0.758	1.213	0.341	0.238
0.7	Random Prior	3	1	0.5	0.5	0.660 (0.004)	0.691 (0.003)	1.472 (0.016)	0.421 (0.004)	0.028 (0.010)
0.7	Random Prior	2	1	0.5	0.5	0.666 (0.006)	0.697 (0.005)	1.462 (0.020)	0.416 (0.005)	0.047 (0.015)
0.7	Random Prior	1	2	0.5	1	0.678 (0.005)	0.710 (0.004)	2.263 (0.035)	0.467 (0.008)	0.086 (0.014)
0.7	Random Prior (weighted)	3	1	0.5	0.5	0.656 (0.004)	0.687 (0.003)	1.506 (0.019)	0.428 (0.004)	0.014 (0.011)
0.7	Random Prior (weighted)	2	1	0.5	0.5	0.665 (0.005)	0.695 (0.005)	1.476 (0.021)	0.419 (0.007)	0.040 (0.016)
0.7	Random Prior (weighted)	1	2	0.5	1	0.677 (0.003)	0.708 (0.004)	2.282 (0.046)	0.470 (0.007)	0.081 (0.012)
0.7	Top Prior	3	1	0.5	0.5	0.645	0.680	1.556	0.435	−0.006
0.7	Top Prior	2	1	0.5	0.5	0.660	0.693	1.502	0.420	0.035
0.7	Top Prior	1	1	0.5	0.5	0.661	0.695	1.459	0.416	0.041

Supplementary Table S2. Results from the optimization procedure across all training fractions to select the optimal weights for the fusion process. Alongside training fraction (t) and Strategy with accompanying n where applicable, we report the selected weights (α, β, γ), the mean across 10 seeds with ($\pm$ SD) for Macro F1, Accuracy, Log Loss, Brier Score, and relative Accuracy Error Reduction Rate (rAER).

t	Strategy	n	α	β	γ	Macro F1	Accuracy	Log Loss	Brier	rAER
0.1	Baseline	—	—	—	—	0.219	0.263	—	—	0
0.1	Random Known	3	1	0	1	0.317 (0.003)	0.369 (0.003)	3.233 (0.032)	0.874 (0.004)	0.143 (0.004)
0.1	Random Known	2	1	0	1	0.317 (0.007)	0.365 (0.007)	3.291 (0.062)	0.879 (0.010)	0.138 (0.009)
0.1	Random Known	1	1	0	1	0.320 (0.007)	0.363 (0.008)	3.446 (0.067)	0.885 (0.009)	0.135 (0.011)
0.1	Random Known (freq)	3	1	0	1	0.317 (0.004)	0.367 (0.004)	3.217 (0.031)	0.876 (0.004)	0.141 (0.005)
0.1	Random Known (freq)	2	1	0	1	0.316 (0.006)	0.364 (0.006)	3.292 (0.049)	0.880 (0.008)	0.136 (0.009)
0.1	Random Known (freq)	1	1	0	1	0.323 (0.008)	0.368 (0.008)	3.416 (0.093)	0.879 (0.014)	0.142 (0.011)
0.1	Top Unknown	3	0.75	0	0.5	0.225	0.268	3.768	0.891	0.006
0.1	Top Unknown	2	1	0	0.5	0.229	0.273	4.353	0.960	0.013
0.1	Top Unknown	1	0.75	0	0.5	0.232	0.286	3.916	0.905	0.031
0.1	Null Set	—	1.75	0	1.5	0.244	0.296	7.741	1.140	0.044
0.1	Random Unknown	—	2	0	1	0.224 (0.003)	0.267 (0.004)	7.901 (0.077)	1.182 (0.007)	0.004 (0.006)
0.1	Random Unknown (freq)	—	1.5	0	0.75	0.226 (0.006)	0.270 (0.007)	6.132 (0.091)	1.099 (0.012)	0.009 (0.010)
0.1	Highest Entropy	3	1.5	0	0.5	0.220	0.265	5.858	1.090	0.002
0.1	Highest Entropy	2	1.5	0	0.5	0.224	0.268	5.822	1.086	0.006
0.1	Highest Entropy	1	1.5	0	0.5	0.226	0.269	5.857	1.085	0.008
0.1	Lowest Entropy	3	0.75	0	0.5	0.221	0.263	3.797	0.893	0
0.1	Lowest Entropy	2	1	0	0.5	0.229	0.269	4.387	0.966	0.008
0.1	Lowest Entropy	1	0.75	0	0.5	0.233	0.286	3.894	0.904	0.031
0.1	Random Prior	3	1.5	0	0.5	0.219 (0.002)	0.264 (0.002)	5.869 (0.016)	1.089 (0.001)	0.001 (0.002)
0.1	Random Prior	2	1.5	0	0.5	0.220 (0.002)	0.265 (0.002)	5.871 (0.030)	1.088 (0.003)	0.002 (0.003)
0.1	Random Prior	1	1.5	0	0.5	0.221 (0.003)	0.266 (0.002)	5.916 (0.029)	1.090 (0.004)	0.003 (0.003)
0.1	Random Prior (weighted)	3	1.5	0	0.5	0.220 (0.002)	0.264 (0.002)	5.882 (0.021)	1.089 (0.002)	0.001 (0.003)

continued on next page

Supplementary Table S2 — continued from previous page

t	Strategy	n	α	β	γ	Macro F1	Accuracy	Log Loss	Brier	rAER
0.1	Random Prior (weighted)	2	1.5	0	0.5	0.220 (0.003)	0.264 (0.003)	5.901 (0.035)	1.091 (0.003)	0.001 (0.004)
0.1	Random Prior (weighted)	1	1.5	0	0.5	0.222 (0.003)	0.266 (0.004)	5.927 (0.054)	1.091 (0.005)	0.004 (0.005)
0.1	Top Prior	3	1	0	0.5	0.218	0.264	4.393	0.960	0.001
0.1	Top Prior	2	1	0	0.5	0.222	0.263	4.382	0.967	0
0.1	Top Prior	1	1.5	0	1	0.227	0.269	6.054	1.103	0.007
0.3	Baseline	—	—	—	—	0.400	0.463	—	—	0
0.3	Random Known	3	2	0	1.75	0.480 (0.002)	0.540 (0.002)	4.171 (0.023)	0.769 (0.003)	0.143 (0.003)
0.3	Random Known	2	1	0	0.75	0.485 (0.003)	0.543 (0.003)	2.315 (0.017)	0.654 (0.005)	0.148 (0.006)
0.3	Random Known	1	1	0	0.75	0.490 (0.006)	0.545 (0.005)	2.284 (0.043)	0.650 (0.007)	0.152 (0.009)
0.3	Random Known (freq)	3	2	0	2	0.482 (0.002)	0.542 (0.001)	4.122 (0.020)	0.766 (0.002)	0.146 (0.002)
0.3	Random Known (freq)	2	1	0	0.75	0.483 (0.003)	0.541 (0.003)	2.326 (0.015)	0.657 (0.003)	0.145 (0.005)
0.3	Random Known (freq)	1	1.5	0	1.25	0.484 (0.003)	0.541 (0.003)	3.143 (0.036)	0.727 (0.004)	0.144 (0.005)
0.3	Top Unknown	3	1.25	0	1	0.429	0.488	3.527	0.768	0.045
0.3	Top Unknown	2	0.75	0	0.5	0.437	0.500	2.518	0.650	0.068
0.3	Top Unknown	1	1.25	0	1	0.470	0.526	3.204	0.716	0.117
0.3	Null Set	—	1.25	0	1	0.469	0.524	3.077	0.713	0.113
0.3	Random Unknown	—	2	0	1.25	0.428 (0.004)	0.487 (0.004)	5.334 (0.080)	0.851 (0.008)	0.044 (0.007)
0.3	Random Unknown (freq)	—	1.75	0	1	0.427 (0.002)	0.488 (0.004)	4.648 (0.033)	0.830 (0.007)	0.046 (0.007)
0.3	Highest Entropy	3	1	0	0.75	0.402	0.467	3.115	0.738	0.006
0.3	Highest Entropy	2	1	0	0.5	0.418	0.477	2.992	0.722	0.026
0.3	Highest Entropy	1	1.75	0	1.25	0.422	0.480	4.921	0.846	0.031
0.3	Lowest Entropy	3	1.25	0	0.75	0.427	0.489	3.450	0.762	0.049
0.3	Lowest Entropy	2	0.75	0	0.5	0.436	0.498	2.503	0.653	0.065
0.3	Lowest Entropy	1	1.25	0	1	0.476	0.529	3.118	0.712	0.122
0.3	Random Prior	3	1	0	0.5	0.404 (0.002)	0.470 (0.003)	3.084 (0.017)	0.734 (0.004)	0.012 (0.006)
0.3	Random Prior	2	1.5	0	1	0.407 (0.004)	0.474 (0.005)	4.229 (0.057)	0.820 (0.008)	0.019 (0.009)
0.3	Random Prior	1	1.5	0	0.75	0.417 (0.005)	0.483 (0.006)	4.079 (0.073)	0.803 (0.009)	0.036 (0.012)
0.3	Random Prior (weighted)	3	1	0	0.5	0.405 (0.003)	0.468 (0.002)	3.108 (0.024)	0.737 (0.002)	0.008 (0.004)
0.3	Random Prior (weighted)	2	1.5	0	1	0.408 (0.005)	0.472 (0.005)	4.270 (0.057)	0.821 (0.007)	0.016 (0.010)
0.3	Random Prior (weighted)	1	1.5	0	0.75	0.416 (0.004)	0.479 (0.003)	4.135 (0.032)	0.809 (0.004)	0.030 (0.006)
0.3	Top Prior	3	1	0	0.75	0.410	0.470	3.063	0.733	0.012
0.3	Top Prior	2	1	0	0.75	0.425	0.476	2.948	0.722	0.024
0.3	Top Prior	1	1.25	0	0.75	0.431	0.485	3.443	0.765	0.040
0.5	Baseline	—	—	—	—	0.541	0.587	—	—	0
0.5	Random Known	3	1.25	0	1	0.637 (0.001)	0.666 (0.001)	1.868 (0.006)	0.507 (0.001)	0.192 (0.003)
0.5	Random Known	2	1	0	0.75	0.640 (0.002)	0.667 (0.001)	1.591 (0.011)	0.477 (0.002)	0.195 (0.003)
0.5	Random Known	1	1	0	0.75	0.645 (0.003)	0.671 (0.003)	1.582 (0.019)	0.471 (0.004)	0.204 (0.007)
0.5	Random Known (freq)	3	1.25	0	1	0.637 (0.001)	0.665 (0.001)	1.870 (0.006)	0.507 (0.001)	0.190 (0.002)
0.5	Random Known (freq)	2	1	0	0.75	0.640 (0.002)	0.667 (0.001)	1.594 (0.011)	0.478 (0.001)	0.194 (0.003)
0.5	Random Known (freq)	1	1	0	0.75	0.643 (0.003)	0.669 (0.004)	1.586 (0.015)	0.472 (0.004)	0.199 (0.009)
0.5	Top Unknown	3	0.75	0	0.5	0.591	0.629	1.703	0.488	0.102
0.5	Top Unknown	2	1	0	1	0.601	0.642	2.026	0.514	0.134
0.5	Top Unknown	1	0.75	0	0.75	0.635	0.669	1.559	0.448	0.199
0.5	Null Set	—	0.75	0	0.75	0.621	0.658	1.531	0.453	0.173
0.5	Random Unknown	—	1	0	0.5	0.583 (0.004)	0.621 (0.004)	1.991 (0.028)	0.531 (0.006)	0.083 (0.009)
0.5	Random Unknown (freq)	—	1	0	0.75	0.588 (0.005)	0.628 (0.005)	2.045 (0.038)	0.527 (0.006)	0.099 (0.011)
0.5	Highest Entropy	3	0.75	0	0.5	0.550	0.598	1.862	0.515	0.028
0.5	Highest Entropy	2	0.75	0	0.5	0.545	0.596	1.902	0.524	0.022
0.5	Highest Entropy	1	0.75	0	0.5	0.554	0.600	1.879	0.520	0.032
0.5	Lowest Entropy	3	1	0	0.5	0.590	0.625	1.959	0.527	0.092
0.5	Lowest Entropy	2	1	0	1	0.597	0.637	2.050	0.521	0.122

continued on next page

t	Strategy	n	α	β	γ	Macro F1	Accuracy	Log Loss	Brier	rAER
0.5	Lowest Entropy	1	0.75	0	0.75	0.634	0.669	1.558	0.448	0.198
0.5	Random Prior	3	1	0	0.5	0.555 (0.005)	0.599 (0.004)	2.110 (0.029)	0.553 (0.006)	0.030 (0.010)
0.5	Random Prior	2	1	0	0.5	0.560 (0.006)	0.604 (0.005)	2.091 (0.040)	0.548 (0.007)	0.041 (0.013)
0.5	Random Prior	1	1	0	0.5	0.571 (0.006)	0.613 (0.005)	2.052 (0.031)	0.540 (0.006)	0.064 (0.012)
0.5	Random Prior (weighted)	3	1	0	0.5	0.552 (0.004)	0.597 (0.004)	2.120 (0.015)	0.556 (0.006)	0.025 (0.010)
0.5	Random Prior (weighted)	2	1	0	0.5	0.557 (0.004)	0.601 (0.004)	2.099 (0.015)	0.552 (0.005)	0.034 (0.009)
0.5	Random Prior (weighted)	1	1	0	0.5	0.572 (0.006)	0.611 (0.003)	2.059 (0.021)	0.543 (0.004)	0.060 (0.008)
0.5	Top Prior	3	0.75	0	0.5	0.540	0.579	2.036	0.550	−0.020
0.5	Top Prior	2	0.75	0	0.5	0.545	0.583	1.950	0.543	−0.008
0.5	Top Prior	1	1	0	0.5	0.568	0.606	2.065	0.553	0.048
0.7	Baseline	—	—	—	—	0.651	0.682	—	—	0
0.7	Random Known	3	2	2	2	0.726 (0.001)	0.747 (0.001)	1.870 (0.008)	0.414 (0.001)	0.203 (0.002)
0.7	Random Known	2	2	2	2	0.727 (0.001)	0.747 (0.001)	1.874 (0.010)	0.412 (0.002)	0.205 (0.003)
0.7	Random Known	1	1	0.5	1	0.739 (0.002)	0.761 (0.002)	1.075 (0.016)	0.335 (0.004)	0.247 (0.008)
0.7	Random Known (freq)	3	2	2	2	0.727 (0.001)	0.748 (0.001)	1.856 (0.010)	0.412 (0.001)	0.205 (0.003)
0.7	Random Known (freq)	2	2	2	2	0.728 (0.001)	0.748 (0.001)	1.856 (0.012)	0.411 (0.002)	0.206 (0.003)
0.7	Random Known (freq)	1	2	2	2	0.734 (0.002)	0.754 (0.001)	1.813 (0.019)	0.397 (0.003)	0.224 (0.004)
0.7	Top Unknown	3	1	0.75	0.75	0.693	0.721	1.330	0.388	0.120
0.7	Top Unknown	2	0.75	1	0.75	0.700	0.722	1.226	0.365	0.126
0.7	Top Unknown	1	1	0	1	0.734	0.760	1.187	0.335	0.244
0.7	Null Set	—	1	0	1	0.729	0.760	1.165	0.334	0.243
0.7	Random Unknown	—	1.75	0	1.5	0.697 (0.005)	0.725 (0.003)	2.040 (0.043)	0.435 (0.004)	0.135 (0.010)
0.7	Random Unknown (freq)	—	1	0	0.75	0.699 (0.007)	0.727 (0.006)	1.339 (0.047)	0.375 (0.009)	0.142 (0.019)
0.7	Highest Entropy	3	1	0	0.5	0.668	0.701	1.426	0.409	0.060
0.7	Highest Entropy	2	1	0	0.5	0.677	0.706	1.418	0.405	0.073
0.7	Highest Entropy	1	1	0.5	0.5	0.676	0.710	1.403	0.399	0.088
0.7	Lowest Entropy	3	1	0.5	0.75	0.691	0.721	1.321	0.387	0.123
0.7	Lowest Entropy	2	0.75	1	0.75	0.701	0.723	1.215	0.366	0.129
0.7	Lowest Entropy	1	1	0	1	0.731	0.759	1.195	0.336	0.241
0.7	Random Prior	3	1	0	0.5	0.662 (0.005)	0.694 (0.004)	1.452 (0.022)	0.416 (0.006)	0.037 (0.014)
0.7	Random Prior	2	1.5	0	1	0.672 (0.007)	0.704 (0.006)	1.867 (0.020)	0.449 (0.008)	0.067 (0.018)
0.7	Random Prior	1	1.5	0	0.75	0.683 (0.004)	0.713 (0.004)	1.754 (0.023)	0.432 (0.006)	0.098 (0.012)
0.7	Random Prior (weighted)	3	1.25	0	0.5	0.661 (0.003)	0.694 (0.003)	1.632 (0.017)	0.437 (0.004)	0.037 (0.008)
0.7	Random Prior (weighted)	2	1.25	0	0.75	0.666 (0.006)	0.700 (0.005)	1.654 (0.017)	0.434 (0.006)	0.056 (0.015)
0.7	Random Prior (weighted)	1	1.75	0	1	0.677 (0.005)	0.711 (0.004)	2.022 (0.044)	0.451 (0.007)	0.091 (0.013)
0.7	Top Prior	3	1	0	0.25	0.658	0.691	1.452	0.418	0.027
0.7	Top Prior	2	1	0	0.5	0.660	0.693	1.464	0.414	0.034
0.7	Top Prior	1	1	0	0.5	0.668	0.700	1.425	0.408	0.056

Supplementary Table S3. Compact summary of the fusion-weight optimization across training fractions and initialization strategies. For each setting, the optimizer evaluated $|\mathcal{E}|$ candidate weight triplets and retained $|\mathcal{P}|$ non-dominated solutions. Reported weights (α, β, γ) are those selected for each run; n_{seeds} indicates the number of stochastic repeats.

t	Strategy	n	α	β	γ	$\|\mathcal{P}\|$	$\|\mathcal{E}\|$	n_{seeds}
0.1	Random Known	3	1	0	1	3	76	10
0.1	Random Known	2	1	0	1	3	76	10
0.1	Random Known	1	1	0	1	4	78	10
0.1	Random Known (freq)	2	1	0	1	3	76	10
0.1	Random Known (freq)	1	1	0	1	3	82	10
0.1	Null Set	—	1.75	0	1.5	5	90	1
0.1	Random Unknown	—	1.67	0	0.92	6	83	10

continued on next page

Supplementary Table S3 — continued

t	Strategy	n	α	β	γ	$\lvert\mathcal{P}\rvert$	$\lvert\mathcal{E}\rvert$	n_{seeds}
0.1	Random Unknown (freq)	—	1.33	0	0.75	4	92	10
0.3	Random Known	3	2	0	1.75	5	76	10
0.3	Random Known	2	1	0	0.75	4	76	10
0.3	Random Known	1	1	0	0.75	2	78	10
0.3	Random Known (freq)	3	2	0	2	4	76	10
0.3	Random Known (freq)	2	1	0	0.75	3	76	10
0.3	Random Known (freq)	1	1.5	0	1.25	3	82	10
0.3	Top Unknown	3	1.25	0	1	5	78	1
0.3	Top Unknown	2	0.75	0	0.5	2	97	1
0.3	Top Unknown	1	1.25	0	1	4	90	1
0.3	Null Set	—	1.25	0	1	4	90	1
0.3	Random Unknown	—	1.58	0	1.08	5	83	10
0.3	Random Unknown (freq)	—	1.33	0	0.83	4	92	10
0.3	Highest Entropy	3	1	0	0.75	9	92	1
0.3	Highest Entropy	2	1	0	0.5	6	89	1
0.3	Highest Entropy	1	1.75	0	1.25	4	79	1
0.3	Lowest Entropy	3	1.25	0	0.75	4	79	1
0.3	Lowest Entropy	2	0.75	0	0.5	2	97	1
0.3	Lowest Entropy	1	1.25	0	1	4	90	1
0.5	Random Known	3	1.25	0	1	4	76	10
0.5	Random Known	2	1	0	0.75	4	76	10
0.5	Random Known	1	1	0	0.75	2	78	10
0.5	Random Known (freq)	3	1.25	0	1	4	76	10
0.5	Random Known (freq)	2	1	0	0.75	4	76	10
0.5	Random Known (freq)	1	1	0	0.75	2	82	10
0.5	Top Unknown	3	0.75	0	0.5	2	78	1
0.5	Top Unknown	2	1	0	1	3	97	1
0.5	Top Unknown	1	0.75	0	0.75	2	90	1
0.5	Null Set	—	0.75	0	0.75	4	90	1
0.5	Random Unknown	—	0.92	0	0.58	6	83	10
0.5	Random Unknown (freq)	—	0.83	0.17	0.58	3	92	10
0.5	Highest Entropy	3	0.75	0	0.5	4	92	1
0.5	Highest Entropy	2	0.75	0	0.5	7	89	1
0.5	Highest Entropy	1	0.75	0	0.5	5	79	1
0.5	Lowest Entropy	3	1	0	0.5	4	79	1
0.5	Lowest Entropy	2	1	0	1	3	97	1
0.5	Lowest Entropy	1	0.75	0	0.75	2	90	1
0.5	Random Prior	3	1	0	0.5	4	80	10
0.5	Random Prior	2	1	0	0.5	3	75	10
0.5	Random Prior	1	1	0	0.5	3	77	10
0.5	Random Prior (weighted)	3	1	0	0.5	3	79	10
0.5	Random Prior (weighted)	2	1	0	0.5	3	82	10
0.5	Random Prior (weighted)	1	1	0	0.5	3	77	10
0.5	Top Prior	3	0.75	0	0.5	3	87	1
0.5	Top Prior	2	0.75	0	0.5	3	96	1
0.5	Top Prior	1	1	0	0.5	6	92	1
0.7	Random Known	3	2	2	2	6	76	10
0.7	Random Known	2	2	2	2	7	76	10
0.7	Random Known	1	1	0.5	1	3	78	10
0.7	Random Known (freq)	3	2	2	2	6	76	10
0.7	Random Known (freq)	2	2	2	2	8	76	10

continued on next page

Supplementary Table S3 — continued

t	Strategy	n	α	β	γ	$\lvert\mathcal{P}\rvert$	$\lvert\mathcal{E}\rvert$	n_{seeds}
0.7	Random Known (freq)	1	2	2	2	4	82	10
0.7	Top Unknown	3	1	0.75	0.75	5	78	1
0.7	Top Unknown	2	0.75	1	0.75	4	97	1
0.7	Top Unknown	1	1	0	1	5	90	1
0.7	Null Set	—	1	0	1	6	90	1
0.7	Random Unknown	—	1.33	0	1.08	4	83	10
0.7	Random Unknown (freq)	—	1.08	0	0.75	6	92	10
0.7	Highest Entropy	3	1	0	0.5	4	92	1
0.7	Highest Entropy	2	1	0	0.5	6	89	1
0.7	Highest Entropy	1	1	0.5	0.5	5	79	1
0.7	Lowest Entropy	3	1	0.5	0.75	6	79	1
0.7	Lowest Entropy	2	0.75	1	0.75	4	97	1
0.7	Lowest Entropy	1	1	0	1	7	90	1
0.7	Random Prior	3	1	0	0.5	5	80	10
0.7	Random Prior	2	1.5	0	1	3	75	10
0.7	Random Prior	1	1.5	0	0.75	4	77	10
0.7	Random Prior (weighted)	3	1.25	0	0.5	4	79	10
0.7	Random Prior (weighted)	2	1.25	0	0.75	5	82	10
0.7	Random Prior (weighted)	1	1.75	0	1	5	77	10
0.7	Top Prior	3	1	0	0.25	6	87	1
0.7	Top Prior	2	1	0	0.5	4	96	1
0.7	Top Prior	1	1	0	0.5	4	92	1

Supplementary Table S4. Changes in Macro F1 (ΔF1) and Accuracy (ΔAcc) for ablation studies assessing the contribution of each fusion component relative to the ML-only (default, no fusion) baseline at each training fraction t. Fusion weights are shown for interpretability (α: ML weight; β: prior weight; γ: loglift/context weight). For each ablation, α and β were fixed as listed, while γ was selected using the procedure described in Methods. Negative values indicate decreased performance relative to the ML-only baseline. "ML guide" indicates that ML predictions are used only to guide initialization/ordering, not as a fused score contribution.

t	Ablation	α	β	γ	ML-Only F1	ML-Only Acc	ΔF1	ΔAcc
0.1	LogLift only + ML guide (Top Unknown n=1)	0	0	0.5	0.219	0.263	−0.191	−0.203
0.1	ML only (Null set)	1	0	0	0.219	0.263	0	0
0.1	Priors+LogLift + ML guide (Top Unknown n=1)	0	1	1	0.219	0.263	−0.165	−0.167
0.1	Priors+LogLift (no ML; Oracle init)	0	1	2	0.219	0.263	−0.184	−0.216
0.1	Priors+LogLift (no ML; Top Prior n=1)	0	1	0	0.219	0.263	−0.219	−0.240
0.1	Priors only (Null set)	0	1	0	0.219	0.263	−0.219	−0.240
0.3	LogLift only + ML guide (Top Unknown n=1)	0	0	1	0.400	0.463	−0.323	−0.310
0.3	ML only (Null set)	1	0	0	0.400	0.463	0	0
0.3	Priors+LogLift + ML guide (Top Unknown n=1)	0	1	2	0.400	0.463	−0.319	−0.325
0.3	Priors+LogLift (no ML; Oracle init)	0	1	2	0.400	0.463	−0.353	−0.393

continued on next page

Supplementary Table S4 — continued

t	Ablation	α	β	γ	ML-Only F1	ML-Only Acc	ΔF1	ΔAcc
0.3	Priors+LogLift (no ML; Top Prior n=1)	0	1	0	0.400	0.463	−0.400	−0.440
0.3	Priors only (Null set)	0	1	0	0.400	0.463	−0.400	−0.440
0.5	LogLift only + ML guide (Top Unknown n=1)	0	0	1	0.541	0.587	−0.441	−0.421
0.5	ML only (Null set)	1	0	0	0.541	0.587	0	0
0.5	Priors+LogLift + ML guide (Top Unknown n=1)	0	1	2	0.541	0.587	−0.435	−0.429
0.5	Priors+LogLift (no ML; Oracle init)	0	1	2	0.541	0.587	−0.489	−0.505
0.5	Priors+LogLift (no ML; Top Prior n=1)	0	1	0	0.541	0.587	−0.540	−0.562
0.5	Priors only (Null set)	0	1	0	0.541	0.587	−0.540	−0.562
0.7	LogLift only + ML guide (Top Unknown n=1)	0	0	1	0.651	0.682	−0.556	−0.510
0.7	ML only (Null set)	1	0	0	0.651	0.682	0	0
0.7	Priors+LogLift + ML guide (Top Unknown n=1)	0	1	2	0.651	0.682	−0.544	−0.512
0.7	Priors+LogLift (no ML; Oracle init)	0	1	2	0.651	0.682	−0.592	−0.591
0.7	Priors+LogLift (no ML; Top Prior n=1)	0	1	0	0.651	0.682	−0.650	−0.657
0.7	Priors only (Null set)	0	1	0	0.651	0.682	−0.650	−0.657

Supplementary Table S5. Mean macro F1 (± SD) across the four headline initialization strategies (Null set, Random Known (n=1), Random Unknown (n=1), and Top Unknown (n=1)) for all training fractions (t) undergoing stochastic corruption with probability ε across three modes: drop (remove each seed label with probability ε), swap (replace each seed label with probability ε by a label not already present), and add ($\varepsilon|S_0|$ decoy labels). Entries report macro-F1 with repeated runs only for stochastic initialisation strategies (Random Known/Unknown), so reported SD reflects variability due to seed selection; deterministic strategies (Null Set, Top Unknown) yield a single value (one corruption realisation) under fixed artefacts and decision rules. Negative values indicate degradation. Baseline macro-F1 using ML-only classifier: 0.219 at t=0.1; 0.400 at t=0.3; 0.541 at t=0.5; 0.651 at t=0.7.

ε	Null set	Random known	Random unknown	Top unknown
Training fraction $t = 0.1$, Corruption mode: Add				
0	0.232	0.306 ± 0.007	0.145 ± 0.017	0.227
0.1	0.232	0.293 ± 0.008	0.145 ± 0.016	0.213
0.2	0.232	0.285 ± 0.009	0.147 ± 0.016	0.212
0.4	0.232	0.265 ± 0.011	0.150 ± 0.012	0.204
0.6	0.232	0.246 ± 0.011	0.155 ± 0.010	0.203
Training fraction $t = 0.1$, Corruption mode: Drop				
0	0.232	0.306 ± 0.007	0.145 ± 0.017	0.227
0.1	0.232	0.297 ± 0.008	0.153 ± 0.014	0.228
0.2	0.232	0.290 ± 0.008	0.162 ± 0.014	0.231
0.4	0.232	0.275 ± 0.009	0.180 ± 0.015	0.232
0.6	0.232	0.264 ± 0.008	0.199 ± 0.012	0.236

continued on next page

Supplementary Table S5 — continued

ε	Null set	Random known	Random unknown	Top unknown
Training fraction $t = 0.1$, Corruption mode: Swap				
0	0.232	0.306 ± 0.007	0.145 ± 0.017	0.227
0.1	0.232	0.284 ± 0.010	0.140 ± 0.016	0.205
0.2	0.232	0.265 ± 0.013	0.139 ± 0.012	0.208
0.4	0.232	0.227 ± 0.019	0.133 ± 0.011	0.183
0.6	0.232	0.188 ± 0.018	0.124 ± 0.008	0.170
Training fraction $t = 0.3$, Corruption mode: Add				
0	0.466	0.490 ± 0.005	0.390 ± 0.011	0.470
0.1	0.466	0.479 ± 0.006	0.385 ± 0.008	0.458
0.2	0.466	0.470 ± 0.007	0.383 ± 0.009	0.454
0.4	0.466	0.454 ± 0.009	0.384 ± 0.009	0.431
0.6	0.466	0.440 ± 0.008	0.385 ± 0.007	0.434
Training fraction $t = 0.3$, Corruption mode: Drop				
0	0.466	0.490 ± 0.005	0.390 ± 0.011	0.470
0.1	0.466	0.488 ± 0.004	0.396 ± 0.011	0.469
0.2	0.466	0.485 ± 0.005	0.402 ± 0.010	0.469
0.4	0.466	0.481 ± 0.005	0.424 ± 0.007	0.469
0.6	0.466	0.475 ± 0.006	0.438 ± 0.006	0.468
Training fraction $t = 0.3$, Corruption mode: Swap				
0	0.466	0.490 ± 0.005	0.390 ± 0.011	0.470
0.1	0.466	0.470 ± 0.011	0.378 ± 0.009	0.438
0.2	0.466	0.455 ± 0.011	0.373 ± 0.010	0.429
0.4	0.466	0.424 ± 0.016	0.368 ± 0.015	0.396
0.6	0.466	0.395 ± 0.012	0.359 ± 0.013	0.393
Training fraction $t = 0.5$, Corruption mode: Add				
0	0.638	0.648 ± 0.003	0.563 ± 0.005	0.643
0.1	0.638	0.639 ± 0.005	0.559 ± 0.006	0.630
0.2	0.638	0.631 ± 0.006	0.557 ± 0.007	0.620
0.4	0.638	0.615 ± 0.009	0.554 ± 0.006	0.600
0.6	0.638	0.602 ± 0.009	0.550 ± 0.007	0.596
Training fraction $t = 0.5$, Corruption mode: Drop				
0	0.638	0.648 ± 0.003	0.563 ± 0.005	0.643
0.1	0.638	0.647 ± 0.004	0.571 ± 0.006	0.642
0.2	0.638	0.646 ± 0.004	0.578 ± 0.005	0.640
0.4	0.638	0.645 ± 0.005	0.600 ± 0.006	0.640
0.6	0.638	0.642 ± 0.005	0.614 ± 0.007	0.639
Training fraction $t = 0.5$, Corruption mode: Swap				
0	0.638	0.648 ± 0.003	0.563 ± 0.005	0.643
0.1	0.638	0.635 ± 0.006	0.559 ± 0.005	0.627
0.2	0.638	0.625 ± 0.007	0.557 ± 0.006	0.617
0.4	0.638	0.599 ± 0.012	0.554 ± 0.009	0.590
0.6	0.638	0.574 ± 0.013	0.546 ± 0.009	0.580
Training fraction $t = 0.7$, Corruption mode: Add				
0	0.727	0.741 ± 0.003	0.669 ± 0.009	0.735
0.1	0.727	0.732 ± 0.005	0.666 ± 0.011	0.717
0.2	0.727	0.724 ± 0.005	0.662 ± 0.010	0.706

continued on next page

Supplementary Table S5 — continued

ε	Null set	Random known	Random unknown	Top unknown
0.4	0.727	0.710 ± 0.007	0.656 ± 0.009	0.694
0.6	0.727	0.697 ± 0.004	0.653 ± 0.008	0.692
Training fraction $t = 0.7$, Corruption mode: Drop				
0	0.727	0.741 ± 0.003	0.669 ± 0.009	0.735
0.1	0.727	0.738 ± 0.004	0.674 ± 0.009	0.733
0.2	0.727	0.737 ± 0.005	0.681 ± 0.007	0.733
0.4	0.727	0.735 ± 0.003	0.695 ± 0.008	0.732
0.6	0.727	0.732 ± 0.002	0.707 ± 0.007	0.730
Training fraction $t = 0.7$, Corruption mode: Swap				
0	0.727	0.741 ± 0.003	0.669 ± 0.009	0.735
0.1	0.727	0.728 ± 0.006	0.663 ± 0.011	0.712
0.2	0.727	0.717 ± 0.007	0.661 ± 0.009	0.698
0.4	0.727	0.694 ± 0.012	0.653 ± 0.010	0.673
0.6	0.727	0.673 ± 0.011	0.647 ± 0.012	0.662

Supplementary Table S6. Mean precision, recall, and macro F1 across the four headline initialization strategies (Null set, Random Known ($n = \{1, 2, 3\}$), Random Unknown ($n = 1$), and Top Unknown ($n = 1$)) for all training fractions (t). Results reflect initialization strategies under the staged optimization weight-selection procedure. Metrics are shown as mean ($\pm$ SD) across random seeds for stochastic strategies; deterministic strategies evaluated once are reported as a single value.

Strategy	n	Macro Precision	Macro Recall	Macro F1
Training fraction $t = 0.1$				
Baseline	–	0.224	0.293	0.219
Random Known	3	0.355 ± 0.004	0.393 ± 0.003	0.317 ± 0.003
Random Known	2	0.354 ± 0.010	0.390 ± 0.008	0.317 ± 0.007
Random Known	1	0.344 ± 0.010	0.388 ± 0.008	0.320 ± 0.007
Top Unknown	1	0.239	0.313	0.232
Null Set	–	0.258	0.324	0.244
Random Unknown	–	0.226 ± 0.008	0.292 ± 0.005	0.218 ± 0.004
Training fraction $t = 0.3$				
Baseline	–	0.402	0.487	0.400
Random Known	3	0.506 ± 0.003	0.561 ± 0.002	0.480 ± 0.002
Random Known	2	0.505 ± 0.003	0.566 ± 0.004	0.485 ± 0.003
Random Known	1	0.513 ± 0.004	0.570 ± 0.006	0.490 ± 0.006
Top Unknown	1	0.489	0.553	0.470
Null Set	–	0.489	0.551	0.469
Random Unknown	–	0.440 ± 0.009	0.515 ± 0.004	0.428 ± 0.004
Training fraction $t = 0.5$				
Baseline	–	0.549	0.611	0.541
Random Known	3	0.663 ± 0.001	0.700 ± 0.001	0.637 ± 0.001
Random Known	2	0.667 ± 0.002	0.701 ± 0.002	0.640 ± 0.002
Random Known	1	0.671 ± 0.003	0.704 ± 0.002	0.645 ± 0.003
Top Unknown	1	0.648	0.698	0.635
Null Set	–	0.631	0.686	0.621
Random Unknown	–	0.588 ± 0.017	0.642 ± 0.011	0.571 ± 0.014
Training fraction $t = 0.7$				

continued on next page

Supplementary Table S6 — continued

Strategy	n	Macro Precision	Macro Recall	Macro F1
Baseline	–	0.655	0.717	0.651
Random Known	3	0.748 ± 0.001	0.774 ± 0.000	0.726 ± 0.001
Random Known	2	0.749 ± 0.002	0.775 ± 0.001	0.727 ± 0.001
Random Known	1	0.752 ± 0.003	0.784 ± 0.003	0.739 ± 0.002
Top Unknown	1	0.737	0.784	0.734
Null Set	–	0.731	0.784	0.729
Random Unknown	–	0.706 ± 0.007	0.755 ± 0.005	0.697 ± 0.005
